# Structure and Protein-Protein Interactions of Ice Nucleation Proteins Drive Their Activity

**DOI:** 10.3389/fmicb.2022.872306

**Published:** 2022-06-17

**Authors:** Susan Hartmann, Meilee Ling, Lasse S. A. Dreyer, Assaf Zipori, Kai Finster, Sarah Grawe, Lasse Z. Jensen, Stella Borck, Naama Reicher, Taner Drace, Dennis Niedermeier, Nykola C. Jones, Søren V. Hoffmann, Heike Wex, Yinon Rudich, Thomas Boesen, Tina Šantl-Temkiv

**Affiliations:** ^1^Institute for Tropospheric Research, Leipzig, Germany; ^2^Department of Biology, Microbiology Section, Aarhus University, Aarhus, Denmark; ^3^Department of Physics and Astronomy, Stellar Astrophysics Centre, Aarhus University, Aarhus, Denmark; ^4^Department of Molecular Biology and Genetics, Section for Protein Science, Aarhus University, Aarhus, Denmark; ^5^Department of Earth and Planetary Sciences, Weizmann Institute of Science, Rehovot, Israel; ^6^Department of Physics and Astronomy, The Institute for Storage Ring Facilities, Aarhus University, Aarhus, Denmark; ^7^Interdisciplinary Nanoscience Center and Center for Electromicrobiology, Aarhus University, Aarhus, Denmark

**Keywords:** ice-nucleating proteins, protein structure, atmospheric bacteria, protein–protein interactions, protein activity

## Abstract

Microbially-produced ice nucleating proteins (INpro) are unique molecular structures with the highest known catalytic efficiency for ice formation. Airborne microorganisms utilize these proteins to enhance their survival by reducing their atmospheric residence times. INpro also have critical environmental effects including impacts on the atmospheric water cycle, through their role in cloud and precipitation formation, as well as frost damage on crops. INpro are ubiquitously present in the atmosphere where they are emitted from diverse terrestrial and marine environments. Even though bacterial genes encoding INpro have been discovered and sequenced decades ago, the details of how the INpro molecular structure and oligomerization foster their unique ice-nucleation activity remain elusive. Using machine-learning based software AlphaFold 2 and trRosetta, we obtained and analysed the first *ab initio* structural models of full length and truncated versions of bacterial INpro. The modeling revealed a novel beta-helix structure of the INpro central repeat domain responsible for ice nucleation activity. This domain consists of repeated stacks of two beta strands connected by two sharp turns. One beta-strand is decorated with a TxT amino acid sequence motif and the other strand has an SxL[T/I] motif. The core formed between the stacked beta helix-pairs is unusually polar and very distinct from previous INpro models. Using synchrotron radiation circular dichroism, we validated the β-strand content of the central repeat domain in the model. Combining the structural model with functional studies of purified recombinant INpro, electron microscopy and modeling, we further demonstrate that the formation of dimers and higher-order oligomers is key to INpro activity. Using computational docking of the new INpro model based on rigid-body algorithms we could reproduce a previously proposed homodimer structure of the INpro CRD with an interface along a highly conserved tyrosine ladder and show that the dimer model agrees with our functional data. The parallel dimer structure creates a surface where the TxT motif of one monomer aligns with the SxL[T/I] motif of the other monomer widening the surface that interacts with water molecules and therefore enhancing the ice nucleation activity. This work presents a major advance in understanding the molecular foundation for bacterial ice-nucleation activity.

## Introduction

Microbially-produced ice nucleating proteins (INpro) are the most efficient catalysts for ice formation in nature (Kanji et al., 2017). Airborne microorganisms utilize these proteins to enhance their survival by reducing their atmospheric residence times (Amato et al., 2015) and epiphytic microorganisms use them to induce frost damage of plant tissues, which releases nutrients to support growth (Lindow et al., 1982). Once, released into the environment, INpro are long lived and can maintain their activity for at least 30,000 years (Creamean et al., 2020). They are found ubiquitously in terrestrial and marine habitats as well as on microbial, plant, fungal and animal surfaces, which all serve as sources for atmospheric INpro (Šantl-Temkiv et al., 2020, 2022; Huang et al., 2021). INpro are unique in that they can initiate freezing just below 0°C, i.e., close to the melting point of ice (Delort and Amato, 2017; Kanji et al., 2017). While pure water freezes close to −38°C, ice in nature frequently forms close to 0°C (Bühl et al., 2013), which underlines the enormous environmental relevance of INpro, including impact on cloud radiative properties and lifetime and extensive frost damage of crops (Yankofsky et al., 1981; Lindow et al., 1982; Christner et al., 2008; Morris et al., 2011; Costa et al., 2014), as well as their commercial applications, including artificial snow production, cloud seeding, crop protection and food preservation (Ward and DeMott, 1989; Margaritis and Bassi, 1991; Li and Lee, 1995; Skirvin et al., 2000). Currently known microbial sources of INpro are plant-associated bacteria of the genera *Pseudomonas*, *Pantoea* and *Xanthomonas* (Morris et al., 2004; Ling et al., 2021), soil fungi of the genera *Fusarium* (Pouleur et al., 1992) and *Mortierella* (Fröhlich-Nowoisky et al., 2015) and several species of terrestrial and marine Cyanobacteria and microalgae (Tesson et al., 2016; Tesson and Šantl-Temkiv, 2018). Despite the diverse sources of INpro‚ bacterial INpro are the only verified INpro with known amino acid sequences (Wolber et al., 1986) and are therefore widely used as molecular models for INpro. However, for decades bacterial INpro have eluded experimentally based structural analysis which has been attributed to their large size and association with cell membranes. Very recently, a new version of the structure prediction tool AlphaFold was released as the first computational method that has been shown to predict a substantial number of atomic three-dimensional protein structures with high confidence, as validated by comparison to experimental structures. The machine learning (ML) based software is a major improvement in comparison to existing structure prediction algorithms, and is a powerful tool to be used when experimental structures remain elusive (Jumper et al., 2021).

Bacterial INpro are membrane-associated proteins with molecular weights in the range of 110-180 kDa (Wolber et al., 1986). INpro are believed to belong to a family of proteins that contain tandemly repeated, elongated, open structures with a theoretically unlimited number of repeats (Andrade et al., 2001). The central repeat domain (CRD), which is composed of a variable number of 16-amino-acid tandem repeats with the consensus sequence “GYGSTxTAxxxSxL[T/I]A,” constitutes the largest part of the INpro molecule (Green and Warren, 1985; Garnham et al., 2011b). Generally, the functional advantage of repeats has been associated with the enlargement of the available binding surface (Treangen et al., 2009), which is involved in ligand binding (Andrade et al., 2001). In the case of INpro molecules, the active-surface area has been suggested to facilitate binding and ordering of water molecules, and thereby the protein would act as a template for the formation of an ice nucleation embryo and thus induce freezing in the whole volume of water (Schmid et al., 1997; Qiu et al., 2019).

Bacterial INpro show two classes of ice nucleation activity that are characterized by distinct nucleation temperatures (Budke and Koop, 2015). The INpro class C, which nucleate ice between −7°C and −10°C, are frequently observed while INpro class A, which nucleate ice between −2°C and −5°C, are rare. While there is a general consensus that the temperature at which INpro nucleate ice correlates with INpro oligomer size (Govindarajan and Lindow, 1988; Wolber and Warren, 1989; Mueller et al., 1990; Ling et al., 2018) and INpro class A has recently been shown to depend on electrostatic interactions (Lukas et al., 2020), the experimental evidence showing assembly of INpro into oligomers and the underlying mechanism that correlates oligomeric state and ice nucleation activity has yet to be provided.

Despite their multifaceted environmental impacts, only a limited number of modelling- and experimental studies on the structures and interactions of bacterial INpro has been carried out (Garnham et al., 2011b; Vanderveer et al., 2014; Pandey et al., 2016; Hudait et al., 2018; Ling et al., 2018; Zee et al., 2019; Roeters et al., 2020; Lukas et al., 2021; Schwidetzky et al., 2021a). For example, based on a homology modelling study, it has been suggested that each of the 16 amino acids repeats forms a right-handed β-helix with a triangular base and three β-sheet faces, which aligns the threonine-x-threonine motifs (TxT motif) along one face of the β-helix and the serine-x-leucine-threonine/isoleucine (SxL[T/I] motif) along the other face, with each motif forming a short β-strand. According to this model, the two β-sheets defined by the TxT and SxL[T/I] faces are the sites where ice nucleation takes place. The authors furthermore hypothesized that two monomers can dimerize along the conserved tyrosine ladder located in the third β-sheet face of the β-helix. This dimerization was suggested to connect the TxT motif of one monomer with the SxL[T/I] motif of the other monomer, thus extending the surface for ice-nucleation (Garnham et al., 2011b). Founded on the outcome of molecular dynamics simulations using this INpro model (Garnham et al., 2011b), Hudait et al. (2018) suggested that the TxT and SxL[T/I] β-sheets have comparable ice nucleation properties and promote ice formation through an anchored clathrate and ice-like motifs, respectively (Hudait et al., 2018).

Using interface-specific sum frequency generation (SFG) spectroscopy in combination with modelling, Pandey et al. (2016) proposed that the extended domain of aligned TxT motifs enhances ice-nucleation through a hydrophilic-hydrophobic pattern (Pandey et al., 2016). Recently, data from a combined SFG and two-dimensional infrared spectroscopy study (Roeters et al., 2020) were shown to agree better with a newer β-helical INpro structure (Garnham et al., 2011b) over an older β-hairpin model (Kajava and Lindow, 1993). They also showed that the protein imposes order on adjacent water molecules at low temperature (Roeters et al., 2021) but this has been shown insufficient to explain the INpro activity (Lukas et al., 2021). Ling et al. (2018) demonstrated that INpro molecules maintain ice-nucleation capacity despite a 4-fold reduction in the molecular size, and that the ice nucleation temperature decreased as a function of a reduced number of repeats (Ling et al., 2018). In spite of these pioneering studies, we are still in need of experimentally verified insights into the link between the structural and functional properties of the INpro molecules.

In this paper we report on the first experimentally validated *ab initio* model of an INpro from a model strain of *Pseudomonas syringae* R10.79 that was previously isolated from rain (Šantl-Temkiv et al., 2015; Alsved et al., 2018; Ling et al., 2021). We reached our goal by combining *ab initio* modeling of the INpro 3D molecular structure with synchrotron radiation circular dichroism (SRCD) and transmission electron microscopy (TEM) analysis as well as ice nucleation assays and modeling of ice cluster formation based on Classical Nucleation Theory (CNT). Combing these complementing methods, we were able to considerably advance our knowledge of the effect of INpro structure and molecular interactions on their ice nucleation activity.

## Materials and Methods

### Modelling of Initial 16 Repeats of the INpro CRD Mono- and Dimer

The sequence of the first 16 repeats of the INpro CRD domain was used for *ab-initio* structure prediction using AlphaFold with a ML algorithm (Yang et al., 2020). The computations were done on the EMCC high performance computing cluster at the Department of Molecular Biology and Genetics at Aarhus University. The structure prediction was performed with all 5 available models and the predicted TM scoring enabled. The best scoring prediction is presented here, and used for the subsequent docking effort. It was selected based on the scoring by the algorithm, by inspection of the predicted aligned error plots, and by visually inspecting the resulting models.

The sequence of the first 16 repeats of the INpro CRD domain was also used for *ab-initio* modelling with Rosetta as implemented with a deep-learning algorithm on the Rosetta web-server (36). The modeling method was selected as ‘TrRefineRosetta’, and no other input was required.

The modelling of the homodimer was performed using the HADDOCK 2.4 webserver (Van Zundert et al., 2016) for both. The tyrosine ladder on both monomers were marked as ‘active residues’. The docking was run with 50,000 structures for rigid body docking, 400 structures for semi-flexible refinement, and 400 structures for final refinement and analysis. All other settings were left as standard. A separate run was performed without marking the tyrosine ladder as active residues. It also showed the tyrosine ladder to be the dimerization interface (data not shown). All figures of the modelling and measurements of the dimensions were made in The PyMOL Molecular Graphics System (Version 2.0 Schrödinger). The sequence identity plot of the INpro CRD was made using Geneious Prime (Rozen and Skaletsky, 2000).

### Design and Cloning of Truncated INpro Molecules

As the repeat sequences in the different protein constructs display some sequence variation originating from the naturally occurring repeat sequence divergence in the full length INpro, we calculated the mean similarity scores for the repeats when comparing them to the consensus sequence “GYGSTxTAxxxSxL[T/I]A” (Garnham et al., 2011b). The similarity scores were assigned to each amino acid in the repeat according to their biochemical properties (Sneath, 1966). The mean similarity score of single repeats in the CRD ranged from 91.4 to 100% with highly conserved repeats close to the N-terminal and less conserved repeats close to the C-terminal. The difference between the mean similarity score for the different constructs was small and ranged from 94.4 to 98.4% (Supplementary Figure S1). Based on the low deviation in mean similarity score, we conclude that the repeat sequences are highly similar and show negligible divergence for all constructs.

DNA from strain *P. syringae* R10.79 (Šantl-Temkiv et al., 2015; Alsved et al., 2018; Ling et al., 2021) was isolated with PowerSoil DNA Isolation kit (Mobio) according to the manufacturer’s instructions. The products were separated on an 1.5% agarose gel and purified using the Nucleotide removal kit (Qiagen). The *ina* gene sequence was obtained from and an *E. coli* codon-optimized synthetic *ina* gene ligated into vector pET24b (Novagen) produced by Genscript (Ling et al., 2018).

**Figure 1:**
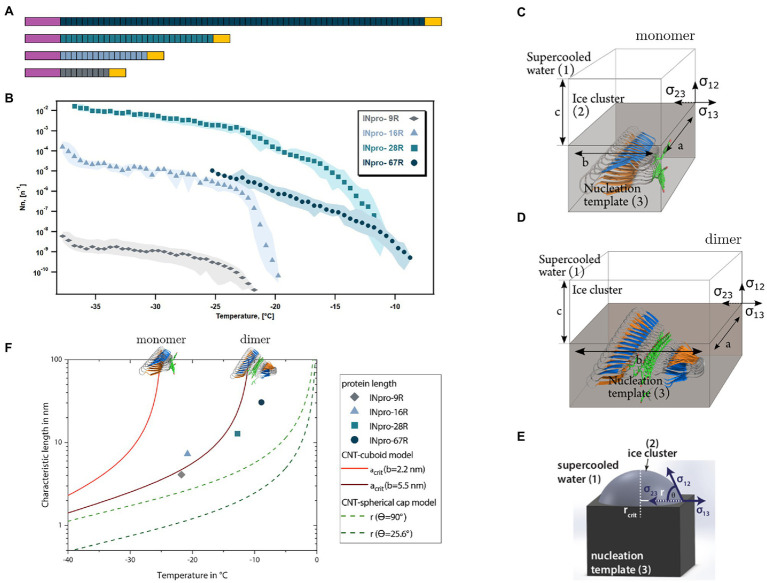
The central repeat region (CRD) is an elongated template for ice nucleation **(A)** Four INpro constructs were produced to investigate the role of CRD size for the nucleation activity. Purple signifies the N-terminal domain and the yellow signifies the C-terminal domain. The colors of the CRD are used to differentiate the recombinant INpro constructs. **(B)** Ice nucleation spectra of INpro constructs expressed as ice nucleation site density per molecule $\left(N_{n}\right)$ as a function of temperature. Each color represents a protein of different length. Averages are shown with maximal and minimal values presented as shaded areas. More detailed figures showing the frozen fractions and the ice nucleation spectra of each protein construct can be found in the SI (Supplementary Figures S8, S9). The quality with which the steepness of the increase was captured depends on the concentration of the purified INpro molecules in each sample. **(C,D,E)** A sketch of the ice nucleation template (3) and the ice cluster (2) in supercooled water (1) for cuboid geometry proposed based on the *ab initio* β-helical INpro model **(C,D)** and spherical cap geometry previously used to model INpro activity **(E)**. The interfaces are defined *via* the respective interfacial free energies $\sigma_{\mathrm{ij}}$ of the adjoining phases. The cuboid is determined by three axes (a, b, c), whereas the spherical cap can be described by the critical radius $r_{\mathrm{crit}}$ and the contact angle θ. **(F)** Comparison of the relationship between the characteristic length (a or r in this figure) and the temperature predicted from the cuboid and spherical cap models according to CNT and the experimental measurements of characteristic nucleation temperatures and lengths of INpro dimers determined from the *ab initio* β-helical model of the INpro.

Based on the DNA extracted from *P. syringae* R10.79 or the synthetic *ina* gene, we produced 7 truncated versions of the gene encoding 7 INpro constructs (Supplementary Tables S1, S2; Figure 1A; Supplementary Figure S2). The INpro-9R, INpro-16R and INpro-28R contained both terminal domains and a reduced number of amino acid tandem repeats, i.e., 9, 16 and 28 repeats, respectively. The INpro-16R-ΔN contained only the C-terminal domain and 16 amino acid tandem repeats. The INpro-15R-ΔT contained only 15 amino acid tandem repeats without the two terminal domains. The INpro-1R-N contained one amino acid tandem repeat with the N-terminal domain and the INpro-C contained just the C-terminal domain. Splicing by Overlap Extension polymerase chain reaction (SOE-PCR) was utilized to obtain genes encoding the INpro-9R, INpro-16R, INpro-16R-ΔN and INpro-28R as previously described in detail for the INpro-16R (Ling et al., 2018). A regular PCR was used to obtain genes encoding the INpro-15R-ΔT, INpro-1R-N and INpro-C. All primers are listed in Supplementary Table S1.

The PCR products were inserted into the pET-30 Ek/LIC vector according to manufacturer’s instructions (LIC Kit, Novagen; Supplementary Table S2). The plasmid DNA was isolated using the Gene Jet Plasmid Miniprep Kit (Thermo Scientific) and its sequence was validated by Sanger sequencing (GATC-biotech).

### Large Scale INpro Production

Vectors containing genes encoding the different protein constructs (Supplementary Table S2) were transformed into Rosetta (DE3) cells. A single colony of transformed *E. coli* was used to inoculate 50 mL of LB medium with 30 μg/mL Kanamycin and cultured at 37°C overnight. The culture grown overnight was inoculated into 12 L lysogeny broth (LB) medium containing 30 μg/mL Kanamycin for the large-scale expression. Once the culture reached an optical density of 0.6–0.8 at 600 nm (OD_600_), it was induced with 1 mM isopropyl β-D-1-thiogalactopyranoside (IPTG) at 20°C overnight. Bacterial cells were harvested by centrifugation at 6,000 × *g* for 15 min at 4°C. Cell pellets were suspended in 25 mL LB per liter of harvested culture and centrifuged at 4,690 × *g* for 30 min. Finally, cell pellets were flash-frozen in liquid nitrogen and stored at −80°C until further treatment.

### Protein Purification

To purify INpro-9R, INpro-16R, INpro-16R-ΔN, INpro-15R-ΔT, INpro-28R, INpro-1R-N and INpro-C, cell pellets were resuspended in 2 mL of lysis buffer 20 mM sodium phosphate, 0.5 M NaCl, 20 mM imidazole; pH 7.4 with a protease inhibitor tablet (Roche) per 1 g of wet cell pellets. Cells were lysed either by 2 rounds of sonication on ice for 5 min (3 cycles at power setting between 70–75%) or with a C5 high-pressure homogenizer (Avestin). During expression tests of INpro-9R, INpro-16R, INpro-16R-ΔN, INpro-15R-ΔT, INpro-1R-N and INpro-C, the proteins were found in both the soluble and insoluble fraction of the cell lysates. For convenience, subsequent work was based on INpro purified from the soluble fractions. Thus, the cell debris and unbroken cells were removed by centrifugation at 12,440 × *g* for 30 min at 4°C and supernatant was filtered through a 0.45 μm filter. In contrast, INpro-28R was only found in the insoluble fraction. Thus, the cell lysate was spun down for 15 min at 1000 × g, for another 15 min at 15000 × *g* and finally at 42,000 rpm for 2 h. The membrane was scraped off and resuspended in solubilization buffer (50 mM Tris HCl pH 8.0, 300 mM NaCl, 5 mM MgCl_2_, 30% glycerol) using a glass homogenizer.

The solution containing the proteins was loaded on a prepacked 1 mL or 5 mL His Trap HP Nickel column (GE Healthcare), which had been washed with 5 column volumes of water and equilibrated with 5 column volumes of buffer A (20 mM sodium phosphate, 0.5 M NaCl, 20 mM imidazole; pH 7.4). After washing the column with 10 column volumes of buffer A, the protein sample was eluted on an ÄKTA Start chromatography system with a gradient from 0 to 100% buffer B (20 mM sodium phosphate, 0.5 M NaCl, 500 mM imidazole; pH 7.4) over 10 column volumes at a flow-rate of 5 mL/min and collected in 5 mL fractions. The protein content of the load, the flow through and fractions of elutes were analyzed on a 12% polyacrylamide SDS-gel. Fractions containing INpro were pooled and for quantification the absorbance was determined at 280 nm using a NanoDrop spectrophotometer. The protein was then concentrated to a final volume of 500 μL using Vivaspin 20 with a molecular weight cut off (MWCO) of 5 kDa (GE Healthcare). The sample was loaded on a Superose6 Increase 10/300 Gl size exclusion column from GE Healthcare that had been equilibrated with size exclusion buffer (for INpro-28R: 20 mM Tris-HCl, 150 mM NaCl; pH 7.6, 0.05% n-Dodecyl β-D-maltoside (DDM); and for INpro-9R, INpro-16R, INpro-16R-ΔN, INpro 15R-ΔT, INpro-1R-N and INpro-C: 20 mM Tris-HCl, 150 mM NaCl; pH 7.6). See an example chromatogram in Supplementary Figure S3. Fractions of 0.5 mL were collected at a flow of 0.2 to 0.4 mL/min and the protein content was analyzed using a 12% polyacrylamide SDS-gel. Fractions containing the INpro were pooled, frozen in liquid nitrogen and stored at −80°C.

The INpro-67R, which was only found in the insoluble fraction, was extracted and purified using a procedure adapted from (Schmid et al., 1997). The harvested cells were suspended in 400 mL of cell resuspension buffer (50 mM Tris-HCl, pH 8.0, 100 mM NaCl, 1 mM EDTA, 1 mM 1,4-dithio-dl-threitol, 3 mg ribonuclease, 3 mg deoxyribonuclease, 5 mM benzamidine, 1 mM phenylmethylsulphonyl fluoride and a protease inhibitor tablet (Roche)) and then disrupted by a Emulsiflex C5 high-pressure homogenizer (Avestin). Intact bacteria and crude membrane pellets were removed by ultracentrifugation at 144 000 × *g* for 1 h at 4°C and the supernatant was diluted 1:1 with 0.2 micrometre filtered milliQ water. An anion-exchange column, HiTrap Q HP column (GE Healthcare), was equilibrated in buffer A (50 mM Tris-HCl pH 8.0, 1 mM EDTA, 1 mM 1,4-dithio-dl-threitol) before loading the protein sample. The flow-through fraction was collected and further purified by ammonium sulfate precipitation. Saturated ammonium sulfate was gradually added to a final concentration of 20% under constant stirring at 4°C. The protein sample was then incubated for 30 min at 4°C and the precipitate was collected at 14 000 × *g* for 10 min at 4°C. The pellet was re-suspended at 100 mg wet weight/mL in buffer B (50 mM Tris-HCl pH 8.0, 1 mM EDTA, 1 mM 1,4-dithio-DL-threitol, 0.1% N-lauroylsarcosine (NLS)) and incubated for 2 h at 25°C. The sample was then centrifuged at 10 000 × *g* for 5 min at 20°C to eliminate undissolved pellet particles. The protein sample was then concentrated to a final volume of 500 μL using Vivaspin20 with a molecular weight cut off (MWCO) of 30 kDa. The final protein purification step and determination of oligomeric state was done on a Superose6 Increase 10/300 size exclusion column (GE Healthcare). The column was equilibrated in buffer B and the concentrated protein was loaded onto the column. Fractions of 0.5 mL were collected and analyzed using SDS-PAGE. Fractions containing the INpro were pooled, frozen in liquid nitrogen and stored at −80°C.

An overview of the protein purification methods used for all constructs is given in Supplementary Table S2.

### Validation of INpro Expression and Purification

INpro expression in *E. coli* cells and the presence of purified INpro in fractions from chromatographic methods was confirmed by SDS-PAGE analysis (e.g., Supplementary Figure S3). Western blot analysis was performed using a primary anti-INpro-1,205 rabbit polyclonal antibody, which targets the residues 1,205–1,220 (CMAGDQSRLTAGKNS) custom-made by Genscript. The blots were probed with a goat anti rabbit secondary antibody (Rockland, United States; 1:5000). Also, Western blots using Anti-His primary antibody (Clontech; 1:4000 dilution) was performed with an anti-mouse HRP conjugate secondary antibody (Dako; 1,3,000) to validate the presence of the protein construct.

### Synchrotron Radiation Circular Dichroism Experiments

SRCD measurements were carried out using the AU-CD beam line of the ASTRID2 synchrotron light source at Aarhus University, Denmark (Miles et al., 2007, 2008). INpro-9R and INpro16R samples were prepared in a 20 mM NaPhos pH 7.5, 150 mM NaF buffer and spectra measured using a nominally 0.1 mm path length cell (quartz Suprasil cell, Hellma GmbH & Co., Germany), with the precise length determined using interferometry measurements (Hoffmann et al., 2016). The concentration of the samples were determined from the absorbance at 205 nm (Anthis and Clore, 2013), which is measured simultaneously with the CD spectrum. Spectra were measured at 25°C in triplicate in the wavelength range of 170 to 280 nm in steps of 1 nm and a dwell time of 2 s, with a corresponding baseline measured on the buffer alone. SRCD data were deconvoluted using the CDSSTR analysis programme and SMP180 reference datasets at the DichroWeb portal (Whitmore and Wallace, 2004, 2008). The secondary structure content for the 7 repeats representing the difference between the INpro-9R and INpro-16R constructs was calculated based on the SRCD data given the cumulative nature of SRCD data.

### Transmission Electron Microscopy

All EM work was done at the iNANO EM facility (EMBION), Aarhus University. Copper grids with a 400 square mesh were prepared with a 2% Collodion solution and carbon coated using a Leica EM SCD 500. The grids were freshly glow-discharged with a PELCO easiGlow™ Glow Discharge Cleaning System prior to loading them with 5 μL of protein sample, blotting and staining 3-times with 3 μL 2% uranyl formate. Samples dilutions 1:5, 1:10, 1:100 and 1:1000 were applied to the grids. TEM images were acquired at a nominal magnification of 67,000× (pixel size size of 1.67 Å) using a FEI Tecnai Spirit transmission electron microscope with a TWIN lens operating at 120 kV. Images were collected with a Tvips TemCam F416 CMOS camera.

### WISDOM Setup and Sample Handling

Ice nucleation measurements were conducted using the WeIzmann Supercooled Droplets Observation on a Microarray (WISDOM). The setup, validation process and temperature calibration are discussed in details previously (Reicher et al., 2018, 2019; Zipori et al., 2018) thus only a general description is given here.

We used a microfluidic device to suspend droplets (with a diameter of 100 μm) containing the proteins in an oil mixture (2% wt Span 80 in mineral oil). Droplet generation was done using NE-500 Programmable OEM Syringe Pump to inject the oil and the sample simultaneously into the device in separate inlets, allowing the oil to press and cut monodisperse droplets in a four-way junction. Droplet size was controlled by changing the ratio of the sample to oil flow. The droplets’ diameter in this study was about 100 μm. Droplets are then trapped in the device’s chamber array, transferred immediately to a Linkam cooling stage (LTS420) coupled to an optical microscope (Olympus BX51) and a CCD camera. The temperature ramp in this study included three different cooling rates: first, droplets were cooled fast at 20°C min^−1^, from the room temperature, in order to reach fast to the region where the proteins were ice-active. Then, a slower cooling rate of 5°C min^−1^ was applied to reach closer to the onset of the freezing, and finally, slow cooling rate of 1°C min^−1^ to follow accurately the freezing events. Freezing events were detected optically, at the point where the droplet becomes darker due to the light scattering of the ice crystals. After all the droplets were frozen, they were heated fast until the melting point, that was recorded at a rate of 1°C min^−1^.

To retrieve the INpro spectra for a wider range of temperatures, samples were analyzed at several concentrations by serially diluting the sample after each analysis by a factor of 10 or 100. The dilution was performed with the buffer until homogenous freezing was observed. Analysis was repeated at least three times for each concentration for statistical validation. The buffer of the INpro-28R and the INpro-67R contained some detergent that interacted with the WISDOM’s oil so we could not produce drops using the buffer. To overcome this issue, we diluted these samples with deionized water by a factor 5 and 2.5 for the INpro-28R and INpro-67R, respectively.

$N_{n}$ was derived based on the previously developed formulation (Vali, 1971). The ice nucleation site density per molecule is given by:


$$N_{n}\left(T\right)=\frac{-\ln\left(1-f_{\mathrm{ice}}\right)}{c_{m}V_{\mathrm{liquid}}\frac{N_{A}}{M}}$$

With

$N_{n}\left(T\right)$ ice nucleation site density per molecule as a function of temperature.

$f_{\mathrm{ice}}$ ice fraction (or number of frozen liquid samples per total number).

$c_{m}$ sample mass concentration.

$V_{\mathrm{liquid}}$ liquid/droplet volume.

$N_{A}$ Avogadro constant.

*M* molar mass of the respective protein construct.

### LINA Setup and Sample Handling

In order to analyze INpro that are present at low concentration, we performed complementary experiments with a second droplet freezing array LINA (Leipzig Ice Nucleation Array; Grawe et al., 2018), based on a previously described instrument (Budke and Koop, 2015). LINA measurements were carried out with 90 droplets each having a volume of 1 μL. These droplets are around 5×10^5^ larger than droplet volumes used for WISDOM, allowing for the detection of rarer INpro. Droplets were placed in separate compartments on a hydrophobic glass slide on top of a cooled Peltier element. We used a standard cooling rate of 1 K/min, recorded the freezing droplets optically and obtained a temperature resolution of 0.1 K with an uncertainty below 0.18 K (single standard deviation; Hartmann et al., 2019).

Similar as WISDOM measurements, we made droplet freezing experiments of dilution series of the INpro samples with the respective buffer to retrieve INpro spectra for a wide range of temperatures until we observed a levelling off indicating that all existing INpro were already activated. $N_{n}$ was derived in the same way as for the WISDOM measurements (Vali, 1971).

### The Characteristic Nucleation Temperature (T_char,50_)

In general, the freezing probability of a droplet population depends on the properties and number of immersed ice nucleating particles (INP). Consequently, within certain limits determined by the INP properties, the higher the number of immersed INP the higher is the temperature range at which the freezing is observed (Augustin et al., 2013; Hartmann et al., 2013; Peckhaus et al., 2016). In order to define a characteristic nucleation temperature, independently of the number of INP, which in our case are INpro, the related calculations are done based on the ice nucleation number site density $N_{n}$ . In some cases, the ice nucleation spectrum exhibits a clear plateau (Hartmann et al., 2013; Niedermeier et al., 2015; Peckhaus et al., 2016). This is indicative of the fact that there are no further INP in the sample which nucleate ice at the respective temperatures for which the plateau shows up. Hence from this, the maximum number of INP of the respective freezing mode can be derived. In this study, instead of observing a clear plateau, we observed that $N_{n}$ continued to slightly increase with decreasing temperature (see e.g., Figure 1B). We assume this to be due to eventually existing degradation products of INpro constructs, which are less efficient in nucleating ice. However, as we do not have such a clear plateau, we determined the knee point of the $N_{n}$ spectra, which we assume to be the maximum number of INpro. The knee point or the knee of a curve describes the point at which the slope of the curve changes. We identified the knee points by using an open-source python function kneed which uses temperature bin averaged logarithmic ice nucleation spectra as input data (Supplementary Figure S4; Supplementary Table S3).^1^ We tested different sensitivity parameters (*S* values) and found that *S* = 1 generally fitted best (data not shown). For consistency, we decided to use *S* = 1 for all calculations. Based on this knee point, the characteristic nucleation temperature (T_char,50_) is defined as the temperature at which the value of $N_{n}$ is 50% of $N_{n}$ at the knee point. The $N_{n}$ at the knee point ranged roughly from 10^−10^ to 10^−5^ ice-nucleation active INpro molecules per total number of INpro molecules.

### Theoretical Description of Elongated Ice Cluster Templates Using Classical Nucleation Theory

Considering heterogeneous ice nucleation, the most commonly used geometry of ice clusters on a flat surface is the spherical cap in CNT (Lamb and Verlinde, 2011; Eickhoff et al., 2019). Ice nucleating particles providing these surfaces for ice cluster formation might have special features that require other geometries, such as hexagonal shape, curved surfaces, pores, completely covered or elongated templates to be described (Fletcher, 1959; Burke and Lindow, 1990; Qian, 2007; Marcolli, 2017; Qiu et al., 2019).

As the shape of the CRD region of the analyzed proteins is much more elongated than wide, we consider these elongated templates for ice nucleation. We assume that the relevant surface area for ice nucleation is characterized by a long and a short axis. We model a simple cuboidal ice cluster which forms at the surface of the elongated ice cluster template (Figures 1C,D). The cuboid has three axes: a represents the longest axis, b the width and c the height.

To form an ice cluster (2, ice) in a metastable parent phase (1, supercooled water) on a template (3, protein structure) energy is released by forming a volume V and consumed to form a surface at the ij and a line at the ijk interfaces. The Gibbs free energy difference between the liquid and the ice phase ΔG assuming the cuboidal geometry of the ice cluster can be written as:


(1) $$\begin{array}{l} \Delta G\left(T\right)=abc\Delta G_{V}+\sigma_{12}\left(2ac+2bc+ab\right)\\ +\sigma_{23}ab+2b\tau_{123}+2a\tau_{123} \end{array}$$

with the respective interfacial free energies $\sigma_{ij}$ , line tensions $\tau_{ijk}$ and $\Delta G_{V}\left(T\right)=-kT/v_{ice}\left(T\right)\ln\left(S_{12}\right)$ including with Boltzmann’s constant $k=1.38065\times 10^{-23}$ m^2^kgs^−2^ K^−1^, the ratio of saturation pressure of liquid water to ice $S_{12}$ and the molecular volume of ice $v_{ice}$ (parameterizations used are given in (Zobrist et al., 2006, 2007)).

As the width of the template and therefore the width of the ice cluster b is limited by the properties of the INpro for this special geometry, we can justify the assumption b=const. In order to derive the critical size of the ice cluster, Eq. (1) is differentiated with respect to c and set to zero. We obtain the critical length $a_{crit}$ with


(2) $$a_{crit}=\frac{-2b\sigma_{12}}{\Delta G_{V}b+2\sigma_{12}}$$

Thus, we obtain the critical height of the cuboidal ice cluster. As a result, the critical length $a_{crit}$ and height (not shown) are independent of each other, but depend both on the constant width b of the cuboid. In the framework of the cuboidal model we consider the critical length $a_{crit}$ as characteristic length as it defines basically the interface area between ice cluster and template.

In the model using the spherical cap geometry (Figure 1E), the ice cluster can be described by the critical radius $r_{crit}=\frac{-2\sigma_{12}}{\Delta G_{V}}.$ Following this approach, the radius of the interface circular area between ice cluster and nucleation template $r=r_{crit}\times sin\theta$ determines the characteristic length (Figure 1E).

In Figure 1F the characteristic lengths of cuboid and spherical cap model as functions of temperature are shown. In general, the higher the temperature the larger is the characteristic length needed to form critical ice clusters in both models. Further the characteristic lengths derived from the cuboidal model are always larger, i.e., energetically less efficient, compared to the spherical cap model. For very high widths b, $a_{crit}$ converges to $r_{crit}.$

At this point we would like to discuss the simplified assumption of a cuboid geometry as a real ice cluster would never take such a shape. Probably a more realistic shape would be an ellipsoidal cap in analogy to the spherical cap or a cuboid with rounded off edges (Qiu et al., 2019). However, the relevant characteristic length is expected to be very similar in assuming these geometries. Consequently, we refrain from considering these much more complicated approaches.

## Results and Discussion

### The INpro Repeats Form a β-helix Structure

**Figure 2:**
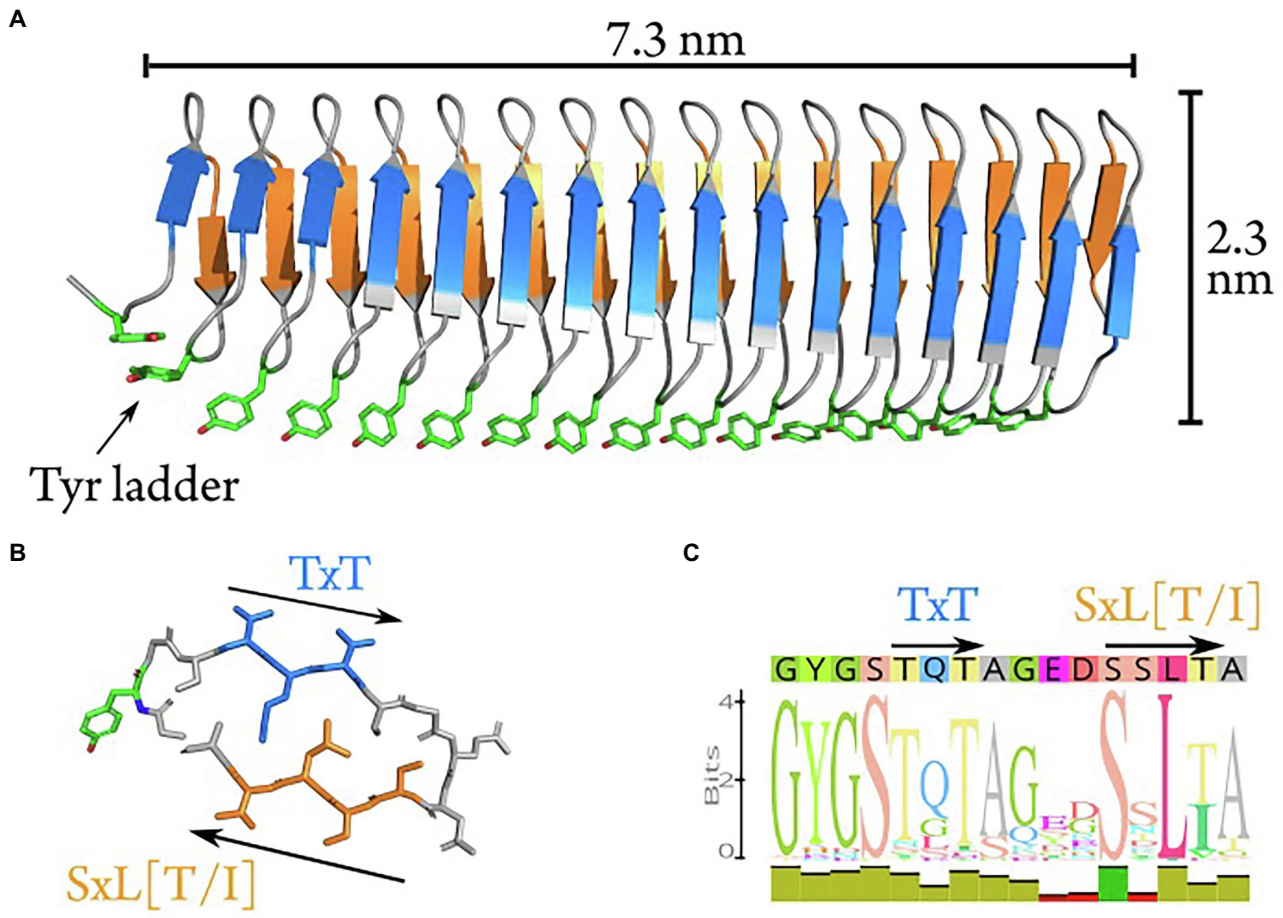
*Ab initio* model of the first 16 repeats of the INpro CRD. **(A)** The *ab initio* model predicted using machine-learning-based algorithms in AlphaFold. The model consists of a β-helix with one extended β-sheet on each side. The β-strands have a rotation along the longitudinal axis of approximately 40 degrees when comparing N- to C-terminal. The highly conserved tyrosine ladder (annotated) is solvent-exposed along the side (shown with stick-representation). **(B)** Stick representation of repeat 13 which displays the best match to the consensus sequence (shown in **C**). The two putative ice-nucleation active sites are annotated as TxT and SxL[T/I], respectively. **(C)** The consensus sequence of the INpro CRD. The predicted structural features are annotated above the sequence corresponding to the color scheme in **(A)** and **(B)**.

Combining ML-based structure predictions of AlphaFold 2 (Jumper et al., 2021) and trRosetta (Yang et al., 2020) with synchrotron radiation circular dichroism data, we propose a new model for the structure based on the initial 16 repeats of the INpro CRD from *P. syringae* R10.79 (Šantl-Temkiv et al., 2015). The model was based on the initial 16 N-terminal repeats, as this repeat section displays the greatest sequence conservation across repeats. The proposed model (Figure 2A; Supplementary Figure S5) consists of two β-strands and two sharp turns for each repeat. The two β-strands form opposing parallel β-sheets. By interactions with neighboring repeats a β-helix structure is formed with an unusual polar core between the β-sheets. The two strands have a limited hydrophobic core consisting of inward facing leucine and alanine residues. Sharp turns are facilitated by the conserved glycine residues in the repeat sequence, and are stabilized by two internal serine ladders – one in each turn (Figure 2B). This differentiates our model from the earlier homology model proposed by Garnham et al. (2011b), which produces a wider loop-like structure in the tyrosine turn forming a third β-strand with no internal serine ladder. Despite these differences, both models share the solvent-exposed tyrosine ladder on one of the turns. The tyrosine residues are fully conserved in the first 59 repeats of the CRD. One β-sheet is decorated with the proposed TxT ice-nucleating motif while the other β-sheet carries the proposed SxL[T/I] ice-nucleating motif. Both β-strands in the repeat form a locally flat surface, but the β-helix itself has a rotation along the longitudinal axis of approx. 45 degrees from N- to C-terminal. The two putative ice-nucleating motifs are pointing in opposite directions relative to the longitudinal β-helix axis. The consensus sequence of the CRD is shown in Figure 2C with annotations of predicted structural features. The model of the complete INpro having 67 repeats and short N- and C-terminal domains is presented in Supplementary Figure S6 and exhibits very similar features as the model of the initial 16 N-terminal repeats. In addition, we employed the trRosetta deep-learning algorithm for parallel *ab initio* modelling of the initial 16 N-terminal repeats and this showed very similar results, albeit with the absence of rotation along the longitudinal axis (Supplemenary Figure S7).

Using the INpro sequence of *P. syringae* R10.79 (Šantl-Temkiv et al., 2015) with a total number of 67 repeats, we designed and produced four recombinant INpro constructs with 9, 16, 28 and 67 tandem repeats in the CRD, i.e., INpro-9R, INpro-16R, INpro-28R and INpro-67R, respectively (Figure 1A; Supplementary Figures S1, S2; Supplementary Table S1). The constructs were expressed in *E. coli*, purified and validated (Supplementary Table S2; Supplementary Figure S3).

**Figure 3:**
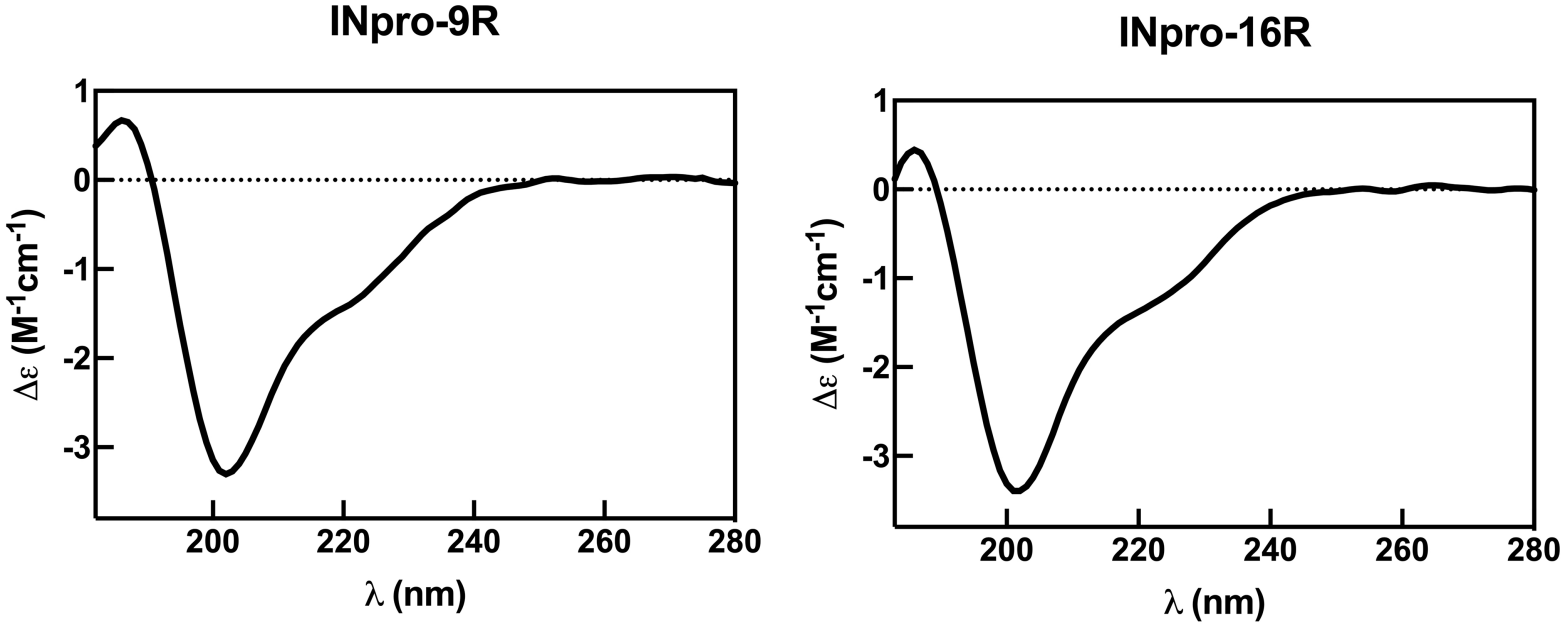
Representative synchrotron radiation circular dichroism spectra for INpro-9R and INpro-16R. The spectra show beta strand/coil/turn structure features when deconvoluted with the SMP180 reference datasets at the DichroWeb portal. Data are shown for the wavelength range 182–280 nm.

To validate the new INpro repeat model, we set out to obtain the first experimental secondary structure data of individual repeats based on recombinant proteins having both the N- and C-terminal domains flanking the CRD domain. Purified INpro-9R and INpro-16R samples were analysed using SRCD spectroscopy and data for multiple concentrations of each sample were deconvoluted using the CDSSTR analysis programme and SMP180 reference datasets at the DichroWeb portal (Whitmore and Wallace, 2004, 2008), thereby confirming consistency of the derived secondary structure content (Figure 3). The secondary structure content for the 7 repeats representing the difference between the two constructs was calculated to be ~35% β-strand, the remaining secondary structure being turns and coil structures. This agrees better with the lower β-strand content (~50%) of our *ab initio* model of a two-β-strand β-helix structure than the three-β-strand β-helix model proposed by Garnham et al. (2011a) that has a β-strand content of ~69% (Garnham et al., 2011b). The difference between a secondary content of 35 and 50% corresponds to each of the strands being one amino acid shorter and these amino acids would then be part of the loop/coil structures. It is possible that the reference data sets (based on known structures) used for deconvolution of the circular dichroism data resulted in the assignment of these amino acids to coil/loops instead of β-strands in the unusual INpro β-helix structure.

### Dimerization on INpro Is a Prerequisite to Class-C Ice-Nucleation Activity

We used purified INpro-9R, INpro-16R, INpro-28R and INpro-67R to investigate the role of the CRD’s repeat number for the ice nucleation activity of INpro (Figure 1A; Supplementary Figures S1, S2; Supplementary Tables S1, S2) using the WISDOM setup (Reicher et al., 2018). This setup utilizes nanoliter size droplets and allows ice nucleation measurements down to – 38°C, the temperature of homogeneous freezing of water. Figure 1B (see also Supplementary Figures S8, S9) depicts ice nucleation site density per molecule ( $N_{n})\;$ as a function of temperature, which we termed the ice nucleation spectrum, for the four different INpro constructs. The shift from a steep to a gentle increase in the ice nucleation spectrum was defined as the knee point of the curve. This can best be seen for INpro-16R that has a knee point just below −20°C (Figure 1B; Supplementary Figure S4; Supplementary Table S3). The other constructs show comparable features and this allowed us to identify the knee points for all ice nucleation spectra (Supplementary Figure S4). The steepness of the ice nucleation spectrum depends on the homogeneity of the INpro molecules present in a sample. Therefore, the steep slopes could either be associated with the activity of functional INpro monomers or with the presence of homogeneous oligomers. We used the knee point to define T_char,50_, i.e., the characteristic nucleation temperature of homogeneous INpro at 50% of the concentration observed at the knee point (Supplementary Table S3). The calculated T_char,50_ were − 23.7°C, −21.7°C, −15.8°C and − 11.2°C for INpro-9R, INpro-16R, INpro-28R and INpro-67R, respectively. Our results clearly show that ice nucleation activity scales with the number of repeats (Figure 1B) but not in a linear fashion. Thus, the relative increase in T_char,50_ as a function of repeat number was less pronounced between INpro-28R and INPro-67R than between INpro-9R and INpro-16R or INpro-16R and INpro-28R (Figure 1B). It should be noted that we only investigated the folding of the two shortest constructs using SRCD in order to assess the beta strand content of the CRD and thus do not have experimental evidence for the folding state of the two largest constructs. However, activity was observed for all constructs indicating the presence of folded INpro species in the samples from all constructs.

In previous studies, it was hypothesized that the size of ice nucleation active molecules plays a major role in determining their characteristic nucleation temperature (Govindarajan and Lindow, 1988; Pummer et al., 2015; Ling et al., 2018; Eickhoff et al., 2019; Qiu et al., 2019). To test this hypothesis, we derived the transverse and longitudinal axes of the constructs using our models of the INpro structure. We estimated that while the transverse axis of the INpro monomers is 2.3 nm in all cases, the longitudinal axis of the CRD is 4.1 nm in the INpro-9R monomer, 7.3 nm in the INpro-16R monomer, 12.8 nm in the INpro-28R monomer and 30.6 nm in the INpro-67R monomer (Supplementary Table S3). We based the rectangular template shape catalyzing cuboidal ice cluster formation on the slim, elongated shape of the INpro molecular model (Figures 1C,D). The rectangular template is described by the lengths of its longitudinal and transverse axes. During the formation of ice clusters in supercooled water, energy is released as the volume of the ice cluster increases, while energy is needed to form the interfaces between the ice cluster and the surrounding liquid. A combination of these volume- and surface effects defines the critical energy barrier for the ice cluster formation. Finally, one of the many continuously forming and melting ice clusters overcomes the critical energy barrier and initiates freezing of the whole supercooled water volume. It is energetically favorable that this process takes place at the surface of the nucleation template. From the critical energy barrier, we derive the respective critical length of the longitudinal axis that forms the critical ice cluster (a_crit_, Figure 1C), assuming a constant transverse axis (b).

**Figure 4:**
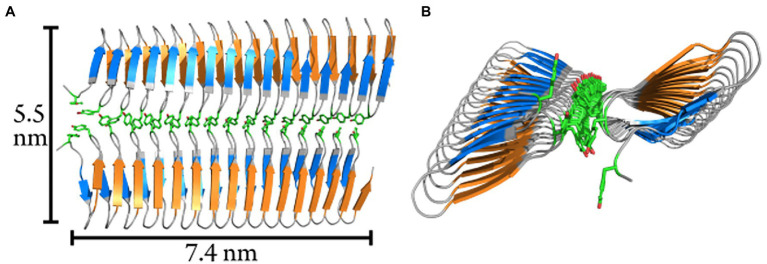
Modelled homodimer structure of the initial 16-repeats of the INpro CRD domain. **(A)** The proposed homodimer structure of the INpro CRD. The tyrosine ladder comprises the dimerization interface. The monomers are parallel, and the TxT motif of one monomer is aligned with the SxL[T/I] motif of another monomer (blue and orange, respectively). The tyrosine ladder forms the dimerization interface. Approximate dimensions of the dimer surface are indicated. **(B)** End-view of the dimer model. The longitudinal rotation in the β-helix causes the dimer to form a saddle-like surface.

Assuming a cuboidal geometry of the ice cluster, we show that the ice nucleation activity is highly width-dependent (Supplementary Figure S10). A single INpro molecule with a width of 2.3 nm is a very poor ice nucleator (Figures 1C,F). For comparison, we also show the standard CNT model that assumes a spherical cap geometry and is also in poor agreement with data from direct experimental measurements of ice nucleation activity of INpro constructs (Figures 1E,F). As the combination of the CNT model and experimental data suggests that the ice-nucleating surface is wider than an INpro monomer (Supplementary Figure S10; Figure 1F), we performed computational docking of the *ab initio* INpro model based on rigid-body algorithms to propose a homodimer structure of the initial 16 repeats of the INpro CRD (see section 2.1). The proposed dimer structure is presented in Figures 4A,B. The monomers align along the highly conserved tyrosine ladder. These result were reproducible for different lengths of the INpro CRD based on AlphaFold 2 modeling (data not shown) as well as for the model of INpro-16R obtained with the trRosetta deep-learning algorithm (Supplementary Figure S11). The dimer model is also in good agreement with earlier molecular dynamics simulations (Garnham et al., 2011b). The suggested dimer model is parallel, meaning that the two monomers are oriented N- to N-terminal, resulting in a surface where the TxT motif of one monomer aligns with the SxL[T/I] motif on the other monomer. During multiple docking runs with varying parameters the parallel dimer model was consistently selected over the anti-parallel by the docking algorithm. The transverse axis of the dimer is 5.5 nm (Figure 4A, TxT and SxL[T/I] marked in blue and orange, respectively), which corresponds very well with the experimentally-measured characteristic nucleation temperatures plotted against the length of the longitudinal axes of the CRD domain for the four INpro constructs (Figures 1D,F). Interestingly, the longitudinal rotation of the β-helix present in each monomer leads to a less flat surface of the dimer when docked along the tyrosine ladder. Instead, the surface adopts a saddle-like structure (Figure 4B). In contrast, the dimer based on the structural model developed by the trRosetta deep-learning algorithm is a flat structure (Supplementary Figure S11). It is not clear how such a saddle-like structure would affect the activity. As INpro is attached to the outer membrane of the bacterial cells, the topology of a dimer and higher-order oligomers will in addition likely be influenced by *in situ* conditions, e.g., membrane curvature assuming that the INpro oligomer is lying flat on the cell surface and interacts with the outer membrane.

In summary, we used CNT-cuboid modelling to investigate whether the predicted INpro dimers would have an activity closer to what we observed with experimental ice nucleation activity measurements compared to monomers. We found out that the experimental data fit very well with the transverse axis length (b-axis) of 5.5 nm estimated for the INpro dimer model (Figures 1D,F). This strongly indicates that the INpro molecules have to form dimers to exhibit observed ice-nucleation activity. We therefore suggest that INpro dimers are responsible for INpro class C ice nucleation.

### Class-A Ice-Nucleation Activity Is Linked to Higher-Order INpro Oligomers

Based on the cuboid model used to obtain data presented in Figure 1F, we conclude that the effect of additional amino-acid repeats in the CRD on the ice nucleation temperature is high when the number of repeats in the CRD is low and decreases as the number of repeats in the CRD is higher. This is due to the fact that the characteristic nucleation temperature, at a certain transverse axis length, is approached asymptotically as a function of increasing number of repeats. This led us to conclude that oligomerization of the INpro dimers into higher order filamentous structures (Garnham et al., 2011a; Zee et al., 2019) raises the characteristic ice nucleation temperature more effectively than increasing the length of a single INpro molecule by adding more repeat modules.

**Figure 5:**
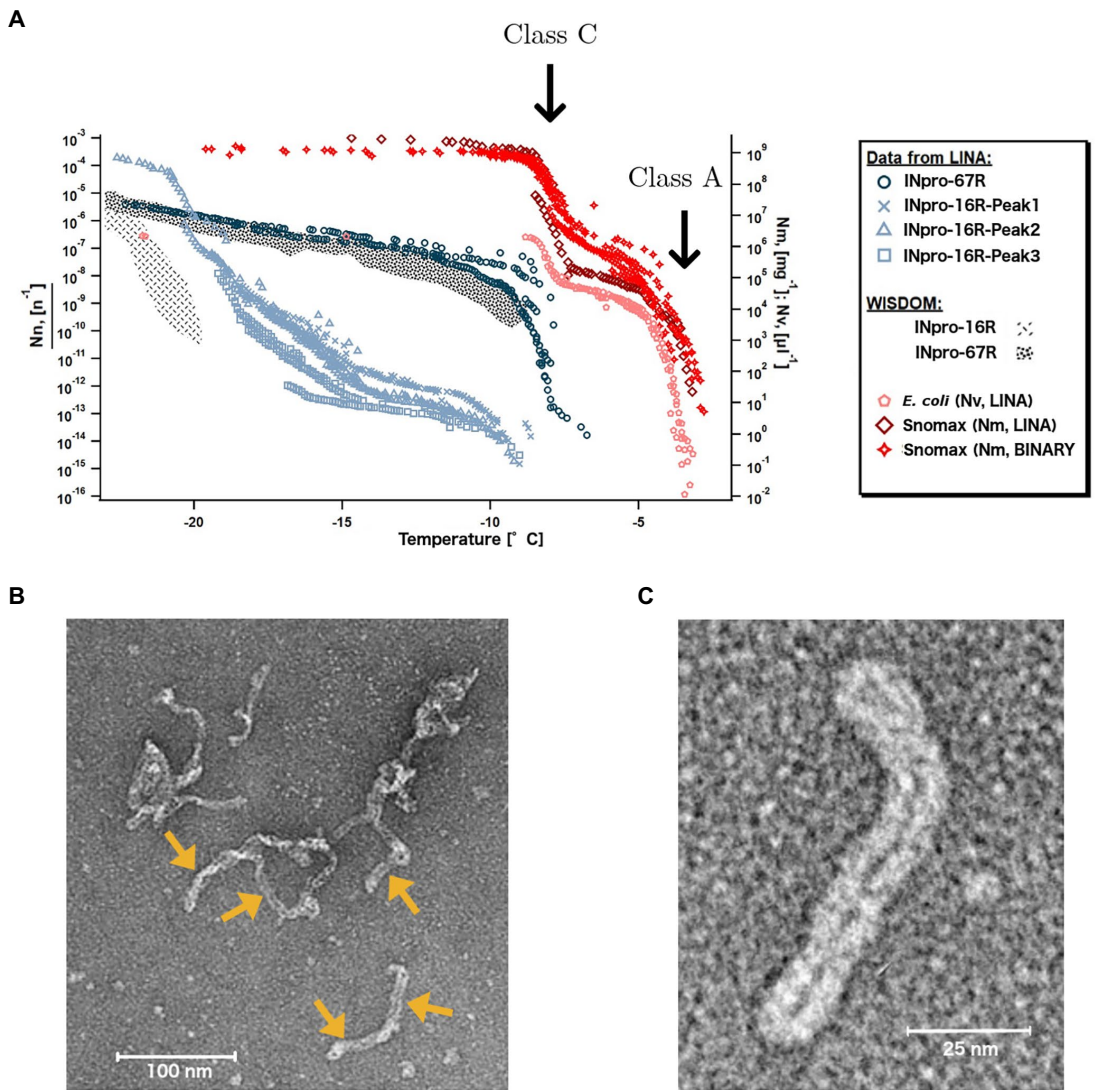
Two types of INpro oligomers are responsible for the ice-nucleation activity of two INpro classes. **(A)** Ice nucleation activity of *E. coli* expressing INpro-67R as well as of purified INpro-67R and INpro-16R proteins measured by the LINA setup. Presumed class A and class C characteristics and structures are indicated for *E. coli* and Snomax® ice nucleation spectra, but are also seen in INpro-16R spectra. Snomax® data were obtained from previous studies (Wex et al., 2015; Knackstedt et al., 2018). Data from WISDOM ice-nucleation measurements of INpro-16R and INpro-67R shown for comparison. **(B)** Representative negative stain TEM image of the INpro-67R showing highly oligomerized, filamentous structures. Individual filamentous structures, marked with yellow arrows, seem to assemble in an end-to-end manner into longer, higher order oligomeric assemblies. **(C)** Representative negative stain TEM image of an individual filamentous structure formed by INpro-67R.

Using the LINA setup (Grawe et al., 2018), which measures ice nucleation using microliter size droplets, we studied whether the less abundant class A INpro activity occurs for different INpro constructs and whether it occurs in the absence of cells and an outer membrane structure. Investigating *E. coli* cells that express INpro-67R, we confirmed the presence of two INpro classes (Figure 5A): class A INpro with T_char,50_ of −3.9°C were ~ 2 orders of magnitude less abundant than class C INpro with T_char,knee_ of −7.6°C (Supplementary Table S3). Our findings are in agreement with recent high-resolution ice-nucleation studies of the same INpro type (Wex et al., 2015; Knackstedt et al., 2018; Figure 5A). The steep slopes observed for the two INpro classes are best explained by two populations of homogenous INpro, while the plateau between the slopes results from the fact that all class A INpro are already activated at a certain temperature (T_char,50_, Supplementary Table S3). For INpro construct with reduced CRD size, we expect that the steep slopes observed for the two classes would occur at lower temperatures. Ice nucleation spectra of purified INpro-16R exhibit two steep slopes, which suggests that this construct exhibits two classes of INpro comparable to the full length protein synthesized by *E. coli* (Figure 5A). The purified INpro-16R sample thus contains two populations of molecular INpro species – a population with a high level of activity (T_char,50_ of −9.3°C, see at > −10°C in Figure 5A, not included in Supplementary Table S3) and another population with a low level of activity (T_char,50_ of −22.2°C, see < −15°C in Figure 5A; Supplementary Table S3) in absence of cells and an outer membrane structure. The ice nucleation spectrum for purified INpro-67R indicates that this may also be the case at higher INpro-67R concentrations, but we were not able to obtain high enough concentrations of purified INpro-67R for activity measurements to fully support this idea. However, based on the presence of two classes of INpro-16R we propose that the activity of class A INpro does not depend on the presence of bacterial cells or an intact outer membrane structure as previously thought (Schmid et al., 1997; Schwidetzky et al., 2021b). Based on these results, we hypothesize that while class C INpro are composed of INpro dimers that induce ice nucleation in the low temperature range, class A INpro are composed of higher order INpro oligomers that induce ice nucleation in the high temperature range. Both INpro classes form in solution from purified proteins in the absence of cells and membranes. We observed a large difference in concentration between the two classes of INpro, which could be attributed either to (i) a higher concentration of INpro dimers compared to INpro oligomers, or to (ii) a concentration-dependent self-assembly of INpro oligomers from the INpro dimers or to (iii) a combination of (i) and (ii) considering a reversible oligomerization process.

During purification, all proteins (INpro-9R, INpro-16R, INpro-28R and INpro-67R) showed multiple peaks in size exclusion chromatography (SEC), e.g., Supplementary Figure 3A. The presence of the correct protein construct in the peak fractions was confirmed with SDS-PAGE (e.g., Supplementary Figure 3B) and Western blot, both demonstrating the presence of different oligomeric protein species in the samples. In all cases, the first peak (INpro-xR-Peak1) eluted close to the void volume of the gel filtration column, indicating the presence of oligomers larger than 700 kDa. This is consistent with the results of Schmid et al. (1997) who showed that the purified INpro molecules had oligomerized (Schmid et al., 1997). The additional peaks appeared after the void volume, indicating the presence of smaller oligomers. Using negative stain TEM, we confirmed that the INpro-xR-Peak1 structures are produced by a highly oligomerized form of the protein, substantially larger than the dimensions predicted by our INpro dimer modeling. INpro-67R-Peak1 structures are shown in Figures 5B,C. These structures assemble into larger elongated, filamentous structures with a twisted, rubber-band-like appearance showing a ~ 6 nm width of the ‘rubber-band’ (double width of ~12 nm for the twisted rubber-band), which fits well with the dimensions of our modeled INpro dimer and is in good agreement with our cuboid model (Figures 1C,D,F). In addition, individual filamentous structures seems to assemble in an end-to-end manner into longer, higher order oligomeric assemblies distinct from amorphous protein aggregates (Figure 5B). Overall, the SEC together with the TEM analysis provided experimental support for the elongated shape of INpro and for the higher-order filamentous oligomers formed from INpro dimers.

### A Putative Role of C-Terminal Domain in Oligomerization

The role of the N-terminal and C-terminal domains for the ice nucleation activity was investigated with WISDOM using four additional protein constructs, i. e. INpro-16R-ΔN (N-terminal domain deleted from INpro-16R construct), INpro-15R-ΔT (15 repeat construct without N- and C-terminals), INpro-N-1R (INpro N-terminal domain with the first CRD repeat), INpro-C (INpro C-terminal domain; Supplementary Figure S2). We observed that the ice nucleation activity of INpro-16R-ΔN was similar to that of INpro-16R that contained an intact N-terminal (Supplementary Figure S12), indicating that the N-terminal is not necessary for dimer formation and thus, does not affect ice nucleation activity of INpro in general. When both terminal domains were removed (INpro-15R-ΔT, 25 kDa), only a monodisperse peak in SEC was observed, with an elution volume roughly corresponding to the molecular weight of the INpro-15R-ΔT monomer based on calibration of the SEC column using globular standard proteins. A monodisperse peak was previously observed in SEC for a similar INpro-15R-ΔT construct, as well as for other INpro constructs that had their terminals removed (Han et al., 2017). In addition, no ice-nucleation activity was observed for INpro-15R-ΔT (Supplementary Figure S12). Based on these results, we conclude that the characteristic nucleation temperature is determined by the CRD size, while the activity depends on the presence of the C-terminal that is involved in INpro oligomerization. It was previously shown that removing the C-terminal from INpro compromised ice nucleation above −5°C (Abe et al., 1989). Han et al., who purified a similar INpro-15R-ΔT, reported that the protein was purified as a monomer and not as a dimer, which would drastically reduce its ice nucleation activity according to the cuboid ice-nucleation model (Figure 1; Han et al., 2017). Our conclusion that the C-terminal of the protein plays a role in protein oligomerization is supported by the observation that the C-terminal itself shows weak but significant ice nucleation activity that is constant and independent of the protein concentration. In contrast, the N-terminal itself shows no ice nucleation activity (Supplementary Figure S12). Cascajo-Castresana et al. recently demonstrated that unspecific protein aggregates can nucleate ice (Cascajo-Castresana et al., 2019). We thus suggest that the small but significant degree of ice nucleation activity by the C-terminal could be associated with the C-terminal domains ability to oligomerize into a larger supramolecular structure as supported by our TEM data, which results in a second ice-nucleation mechanism (active at low subzero temperatures) that is distinct from ice-nucleation initiated by the CRD alone but which may be critical for the INpro assembly into dimers and higher order oligomers.

## Conclusion

We present the first analyses of *ab initio* models of the 3-D structure for full length and truncated versions of INpro obtained with AlphaFold and trRosetta. The proposed model of the INpro CRD consists of repeat units of two interacting β-strands connected by two sharp turns and this was supported experimentally by synchrotron radiation circular dichroism data. Stacking of repeat units results in a β-helix structure with an unusual polar core containing two rows of serine ladders. The two β-sheet faces of the β-helix structure are decorated with the suggested TxT and SxL[T/I] ice-nucleating motifs, respectively. Experimental data on ice nucleation activity of purified INpro constructs were fitted with models based on CNT, which assumed a cuboid shape of INpro derived from the *ab initio* structural model. We thus found that INpro must form dimers to demonstrate observed class C activity. By performing computational docking using rigid-body algorithms we show that a parallel dimer structure based on the *ab initio* INpro model can be assembled through interactions between the highly conserved tyrosine ladder in the CRD as suggested previously based on homology modelling and molecular dynamics (Garnham et al., 2011a). This yields a surface where the TxT motif of one monomer aligns with the SxL[T/I] motif on the other. Using transmission electron microscopy, we show the formation of higher-order filamentous INpro oligomers, which we suggest are associated with class A activity. We show that class A activity is maintained with purified protein constructs in the absence of cells and an intact outer membrane structure, indicating that higher order oligomers self-assemble from INpro dimers. Experimental SRCD data showing the expected beta-strand containing fold for the two shorter INpro conctructs were obtained providing a firm basis for structure and function comparisons whereas for the longer constructs an intact folding state had to be deduced based on the observed ice-nucleation activities of these constructs. As dilution of class A INpro leads to the appearance of class C INpro, as observed by LINA and WISDOM measurements, we suggest that higher-level oligomers form through reversible concentration-dependent self-assembly of dimers. Finally, studies using a C-terminal deficient version of the INpro, allows us to suggest that while the characteristic nucleation temperature depends on the number of amino acid repeats in the CRD, its activity ultimately depends on the presence of the C-terminal that seems to be involved in INpro oligomerization. Overall, this study unravels the role of bacterial INpro shape, size and specific oligomerization state for their ice-nucleation activity and develops an improved theoretical framework of its ice-nucleation activity. Thus, our results form a basis for (i) obtaining a fundamental understanding of ice-nucleation activity in microbial cells; (ii) promoting future molecular dynamics simulation studies by providing a testbed and thus bridging the gap between simulation results and experimental data; (iii) the quantitative understanding of the role of INpro in atmospheric ice formation, by providing an opportunity to adopt a theoretical description of INpro for weather and climate modelling; (iv) enabling commercial manufacturing of ice-nucleating particles including INpro class A for industrial applications such as food preservation and crop protection.

## Data Availability Statement

The original contributions presented in the study are included in the article/Supplementary Material, further inquiries can be directed to the corresponding authors.

## Author Contributions

KF, TB, ML, and TŠ-T designed the research project. ML, TB, and LD designed the protein constructs and optimized the purification. TŠ-T and TB supervised the project. ML, LJ, SB, NR, AZ, SG, TD, and SH performed the experiments. TD, TB, NJ, and SH performed SRCD analysis. LD performed the *ab initio* modeling under supervision of TB. SH and DN performed the CNT modeling. TŠ-T wrote the manuscript with contributions from all authors. All authors contributed to the article and approved the submitted version.

## Funding

This work was supported by the Danish National Research Foundation (DNRF106, to the Stellar Astrophysics Centre, and DNRF136, to the Center for Electromicrobiology, Aarhus University), the AUFF Nova programme (AUFF-E-2015-FLS-9-10), the Villum Fonden (Research grants 23175 and 37435), the Novo Nordisk Foundation (NNF19OC0056963), and the Independent Research Fund Denmark (9145-00001B).

## Conflict of Interest

The authors declare that the research was conducted in the absence of any commercial or financial relationships that could be construed as a potential conflict of interest.

## Publisher’s Note

All claims expressed in this article are solely those of the authors and do not necessarily represent those of their affiliated organizations, or those of the publisher, the editors and the reviewers. Any product that may be evaluated in this article, or claim that may be made by its manufacturer, is not guaranteed or endorsed by the publisher.

## Footnotes

^1^ https://kneed.readthedocs.io/en/stable/
