# Supplementary material for: Structure and Protein-Protein Interactions of Ice Nucleation Proteins Drive Their Activity
Source: Front Microbiol. 2022 Jun 17;13:872306. doi: 10.3389/fmicb.2022.872306 (PMC9247515; doi:10.3389/fmicb.2022.872306)
Supplement: Supplementary file 1 [file Data_Sheet_1.pdf]

Supplementary Material

1 Supplementary Figures and Tables

1.1 Supplementary Figures

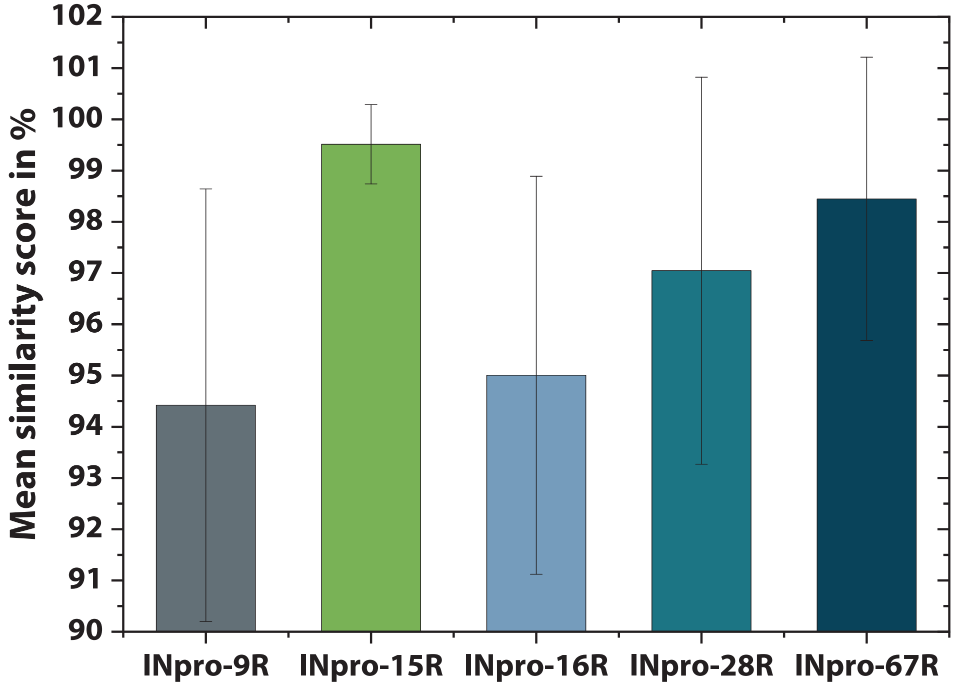

Figure S1. The mean similarity scores of all repeats for the different INpro constructs.

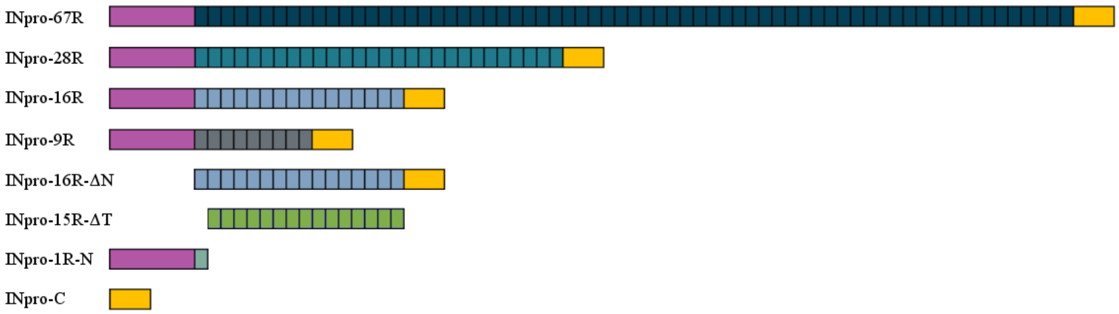

Figure S2. Sketch of all protein constructs used for the study. Purple color signifies the N-terminal domain and the yellow color signifies the C-terminal domain. The blue and green shades in the CRD are symbolic and are used to emphasize the different INpro constructs.

A

B

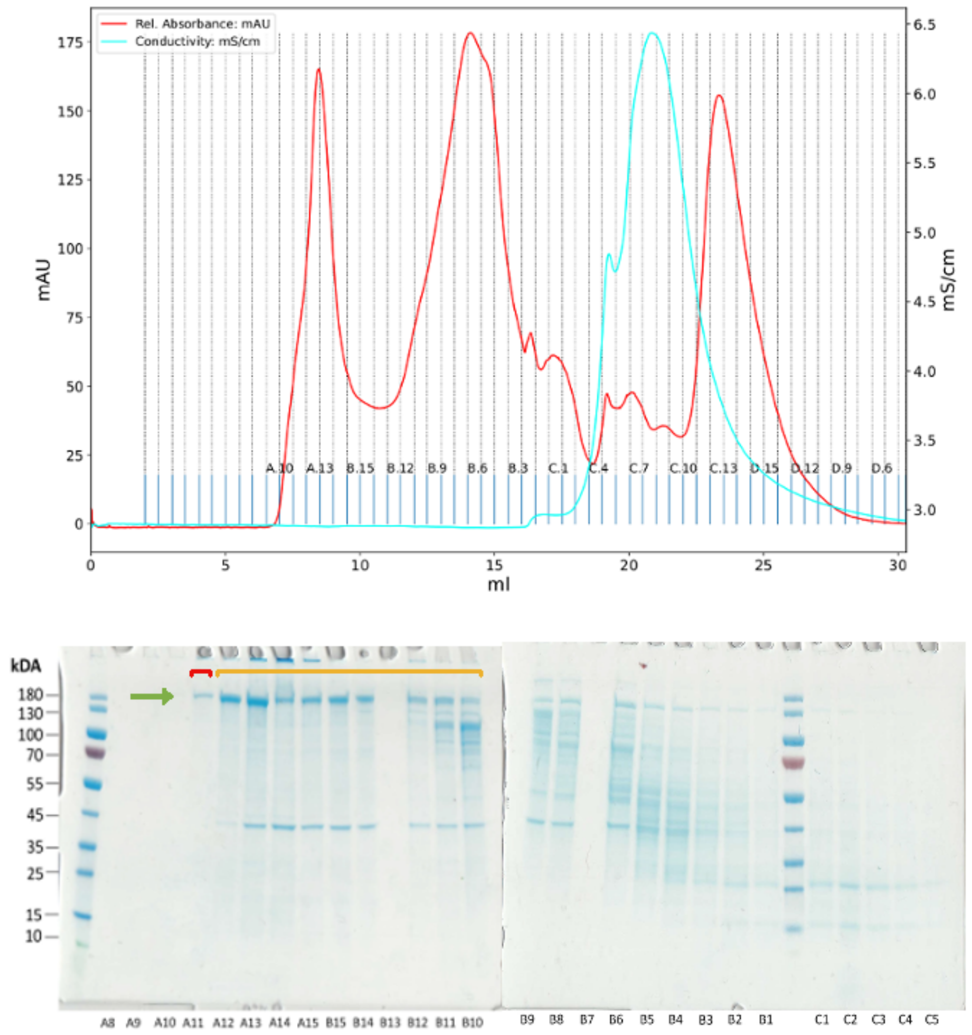

Figure S3. Example size exclusion chromatogram and SDS-PAGE gel image. **(A)** A chromatogram from a size-exclusion chromatography (SEC) of the INpro-67R is shown. Absorbance at 280nm is shown for individual 0.5 mL fractions. **(B)** SDS-PAGE gel for the selected fractions is shown. The green arrow indicates the band corresponding to the INpro-67R. Even though the theoretical molecular weight of INpro-67R was 127.26 kDa the protein migrated just above the 130 kDa marker. Its identity was veryfied by Western blotting using an INpro-specific antibody (data not shown). The first eluted fraction was stored as INpro-67R-Peak1 (shown in red). The following fractions were pooled as INpro-67R-Peak2 (shown in orange).

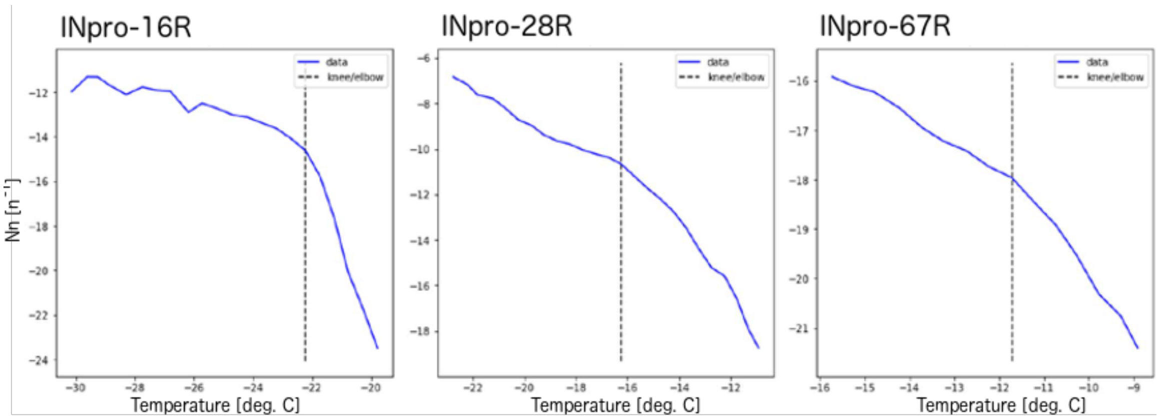

Figure S4. Examples of knee point detection applying the sensitivty parameter 1.0.

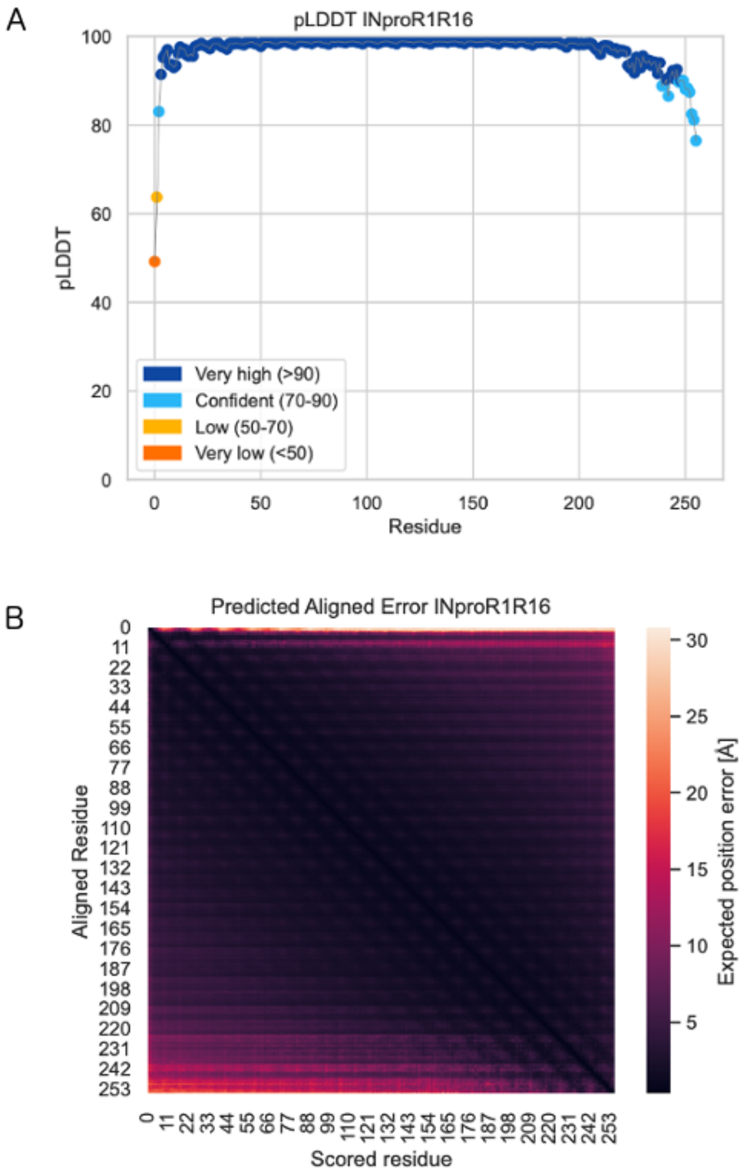

Figure S5. Additional information from Alphafold for the predicted structure of INpro CRD repeat 1 to repeat 16. The structure is presented in the main paper. **(A)** pLDDT score as a function of residue number. The score ranges from 0-100, with higher scores indicating a higher confidence. (B) Predicted aligned error plot. This plot shows the expected position error between pairs of residues. Hereby revealing the confidence in the overall topology of the protein, and the predicted domains. It is shown as residue vs residue. Dark colors show increased confidence.

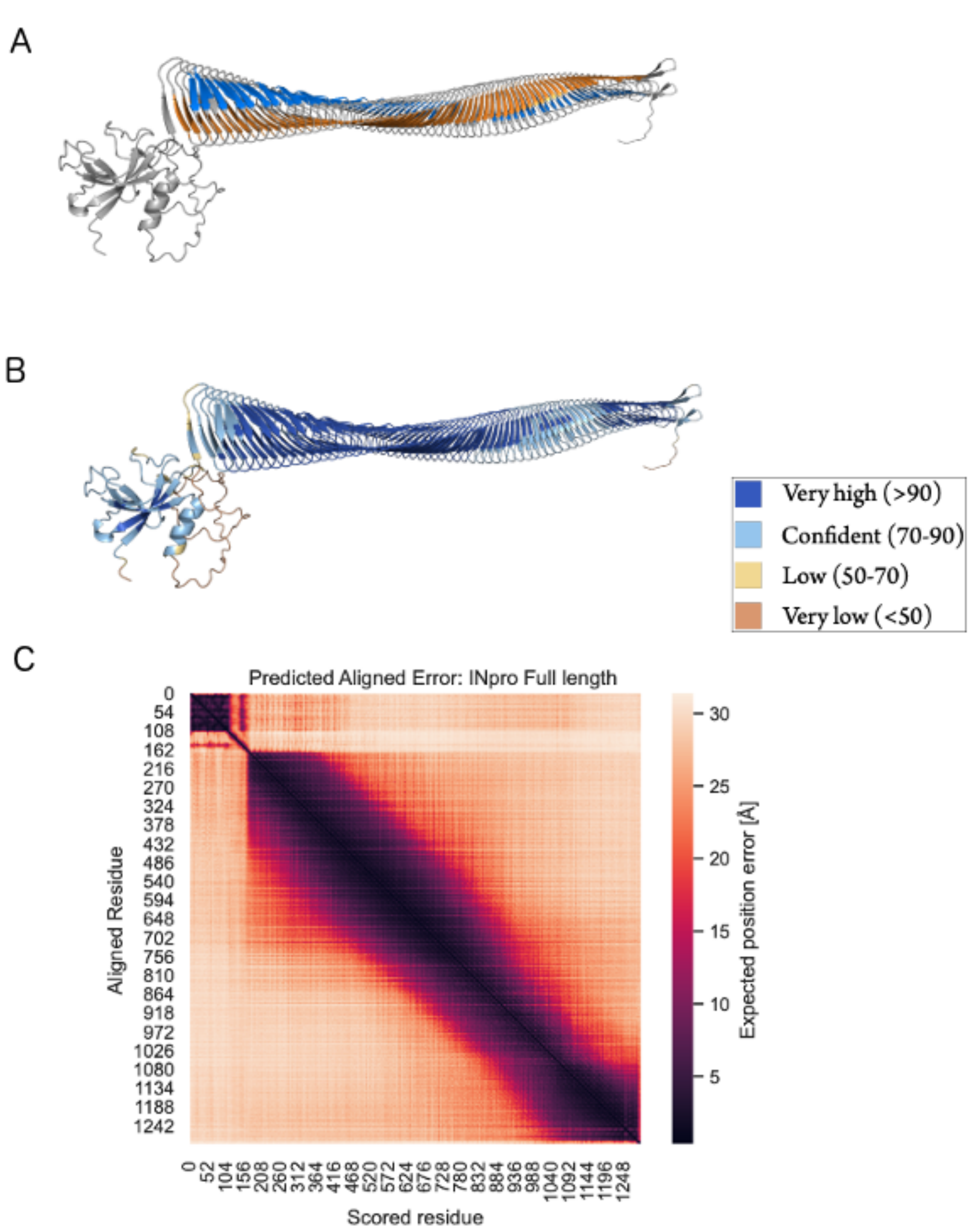

Figure S6. Alphafold structure prediction of INpro-67R. **(A)** The predicted structure colored in accordance with figures in the main paper. Alphafold predicts a folded domain in the N-terminal region (top, left), an unstructured (or of unknown fold) linker, a beta-helix CRD domain, and short capping of the beta-helix by the C-terminal domain ending in an unstructured (or of unknown fold) part. **(B)** Same figure colored according to the Alphafold pLDDT score. The score ranges from 0-100, with higher scores indicating a higher confidence. **(C)** Predicted aligned error plot from Alphafold. This plot shows the expected position error between pairs of residues. Hereby revealing the confidence in the overall topology of the protein, and the predicted domains. It is shown as residue vs residue. Dark colors show increased confidence.

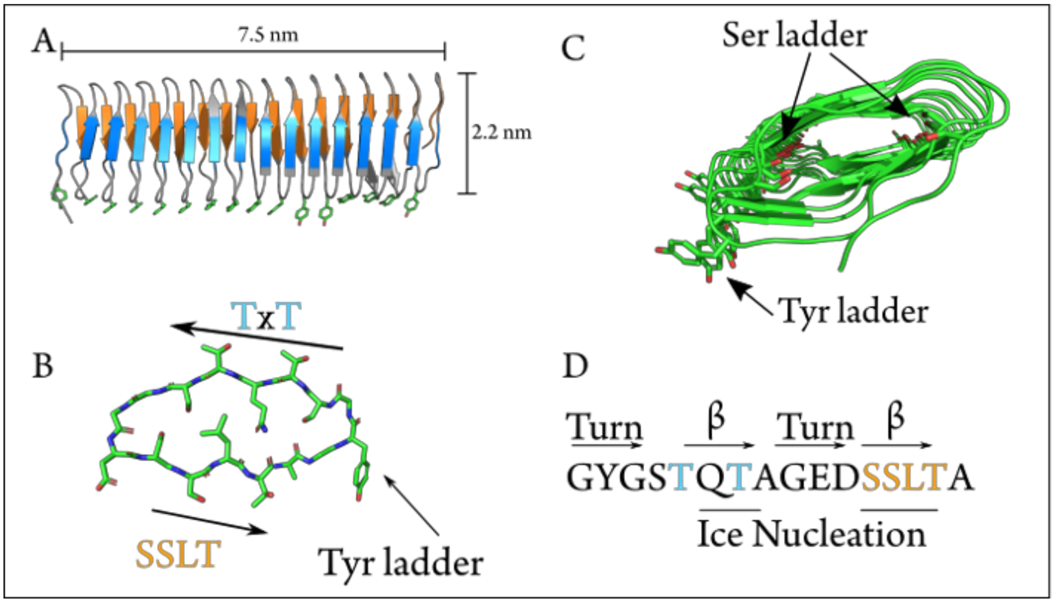

Figure S7. Ab-initio structure of INpro 16-repeat CRD. **(A)** The ab-initio structure determined using deep-learning-based algorithms in trRosetta. The structure consists of two β-helical segments with a modest hydrophobic core. The helical structure is facilitated by the sharp turns promoted by the glycines in the repeat sequence. Aligned along one edge of the structure are the highly conserved tyrosine ladder (shown with stick-representation. **(B)** The β-helical structure seen from the c-terminal end. Closer inspection reveals that each turn is stabilized by an internal serine ladder (annotated and represented as sticks). **(C)** Stick representation of a single repeat matching the consensus sequence (shown in **D**). The two probable ice-nucleation active sites and the tyrosine ladder are annotated. **(D)** The consensus sequence of the initial 16-repeat segment. The predicted structural features are annotated above the sequence, and the probable ice-nucleating sites are marked in red.

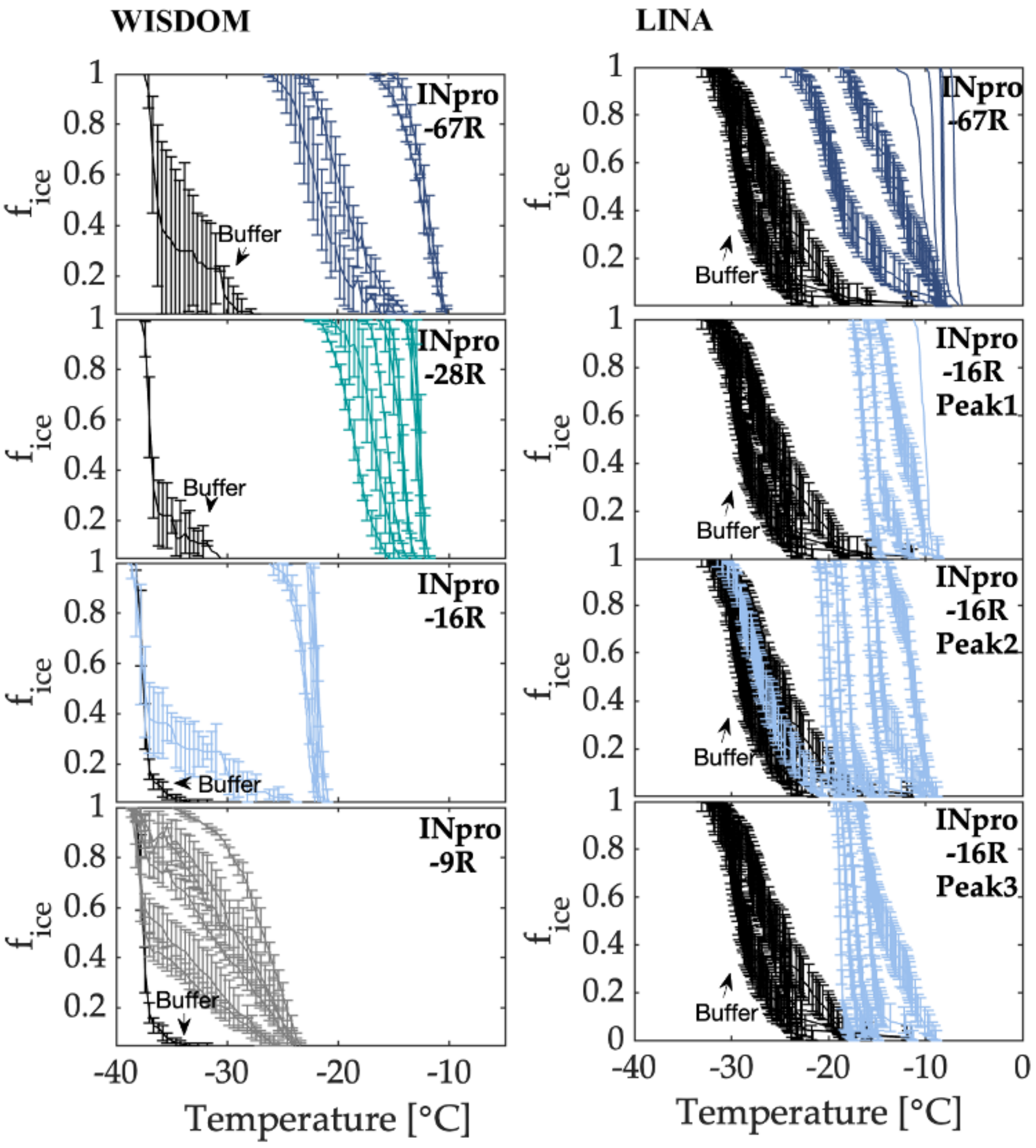

Figure S8. Frozen fractions are shown for INpro-9R, INpro-16R, INpro-28R and INpro-67R as a function of temperature as measured by WISDOM and LINA. Error bars are indicated for all measurements except in cases where they were within the instrument temperature uncertainty.

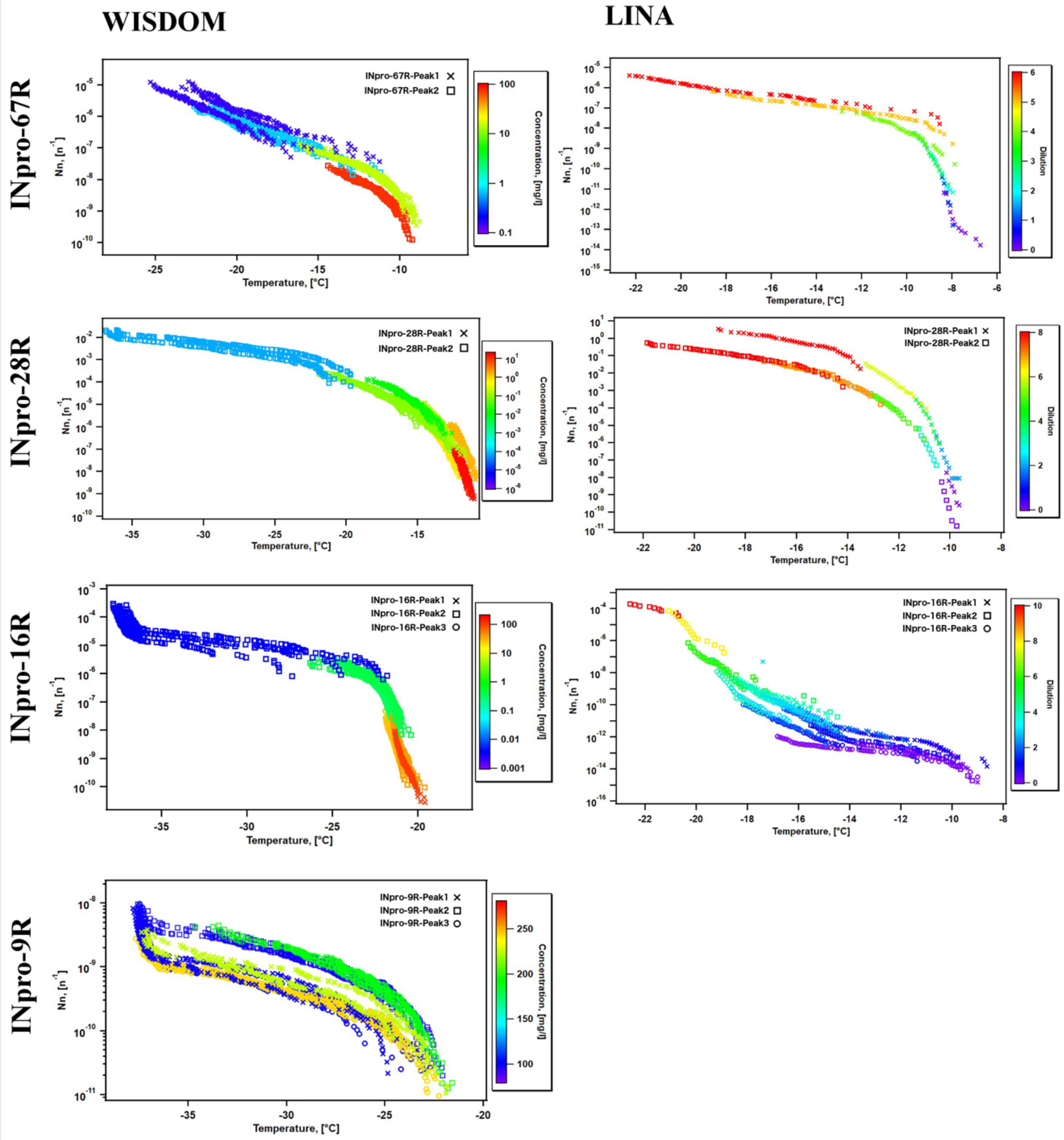

Figure S9. Ice-nucleation spectra for INpro-9R, INpro-16R, INpro-28R and INpro-67R. N_n is shown as a function of temperature as measured by WISDOM and LINA. The different symbols represent different peaks of the same sample (P1, P2, P3). Plots on the left are color coded with respect to concentration and plots on the right with respect to dilution (10-fold dilutions are presented on a logarithmic scale). The fact that N_n at the knee point for the different protein constructs ranged over several orders of magnitude (from 10^{-10} to 10^{-5}) indicated that the difference in purification procedures (18, 63–66) and the stability of the individual constructs had an effect on the ratio between the correctly folded and active INpro molecules and all INpro molecules (or total purified protein mass as measured by the absorbance at 280 nm) in individual samples. The fact that there is still some increase in N_n below the steepest region can be explained by the presence of a heterogeneous population of ice nucleation active compounds, which is likely due to the

presence of INpro degradation products (63–65). Periodic proteolytic degradation of INpro, which decreased the nucleation temperature, has previously been reported for INpro (63–65).

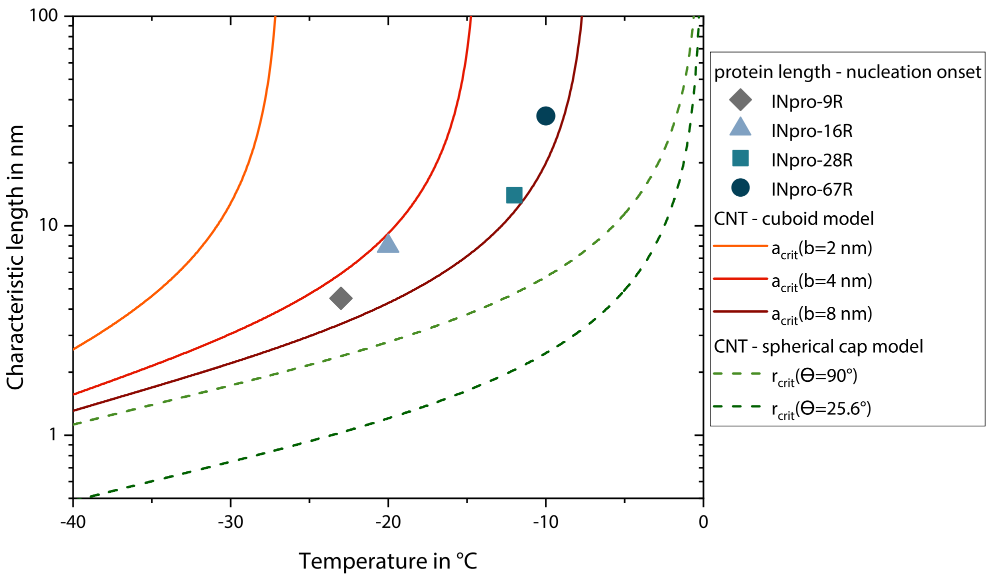

Figure S10. Comparison of the relationship between the characteristic length (a or r in Figures 2C-E) and the temperature predicted from the cuboid and spherical cap models according to CNT.

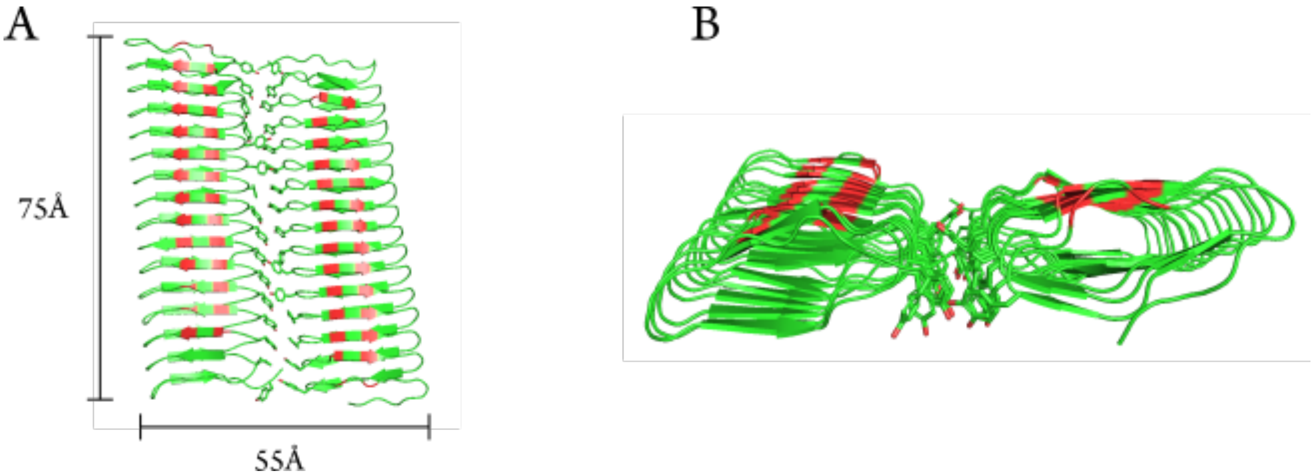

Figure S11. Modelled homodimer structure of the INpro 16-repeat CRD domain based on the trRosetta model. (A) The proposed homodimer structure of the INpro CRD. The tyrosine ladder comprises the dimerization interface. The monomers are anti-parallel, and present a very flat dimer surface exposing the TxT ice-binding motif on the same side

(shown in red). Approximate dimensions of the TxT ice-binding surface of the dimer are indicated . (B) End-view of the dimer model. The tyrosine ladder forms the dimerization interface.

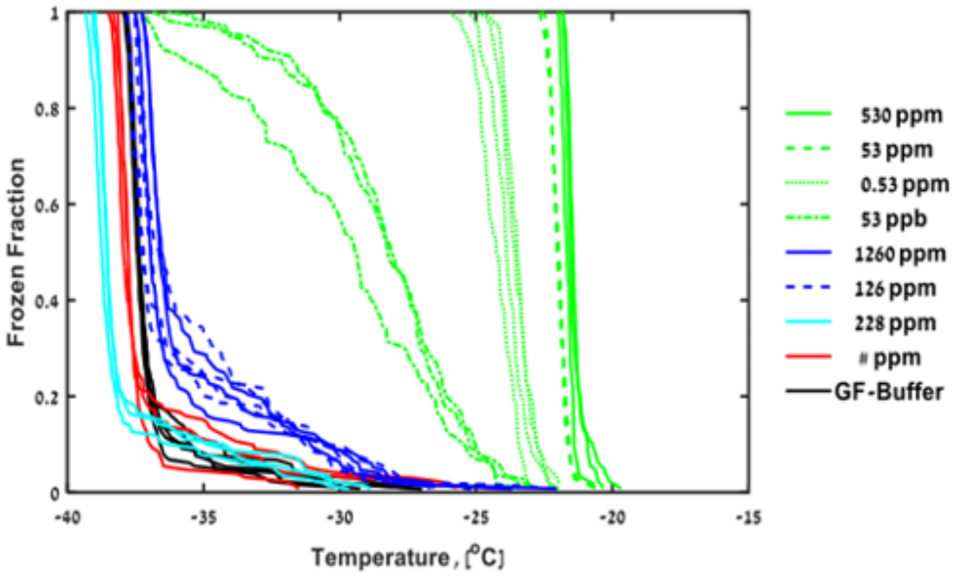

Figure S12. Frozen fraction as a function of temperature for INpro-16R-ΔN (green), INpro-C (blue), INpro-N-1R (cyan) and INpro-15R-ΔT (red) samples measured by WISDOM.

1.2 Supplementary Tables

Table S1. Primers used for different protein constructs.

Table S2. An overview of cloning and purification details for all protein constructs used in the study.

Table S3. Properties of the different protein constructs: presumed state of oligomerization, geometry, INpro class with respective characteristic nucleation temperatures measured with WISDOM and LINA instruments.

Table S1: Primers used for different protein constructs. The underlined sequences correspond to the ligation-independent cloning (LIC) overhangs.

Name	Base pairs	Forward primer	Reverse primer
INpro-16R	1bp-570bp	GACGACGACAAGATGGATTACGACATCCCCACTACTGAGAATCTTTATTTTCAGGGCATGAACCTGGATAAAGCTCTGGT	GATCAGGTCTGAGTGATTGCCCGCGGTTTC
	3019bp-3882bp	GCAGGCTATGGTTCTACGCAAACCGCGGGT	GAGGAGAAGCCCGGTTCACTATTCAACTTCAATCCAATCGTCTTCTT
INpro-9R	1bp-570bp	GACGACGACAAGATGGATTACGACATCCCCACTACTGAGAATCTTTATTTTCAGGGCATGAACCTGGATAAAGCTCTGGT	ACCTGCGGTCTGGTTACTGTCTGCACCTGC-GGTCAGTGCGCTATCACCGCCCGCGGTCTGCGTCGAACCATAGCCAGCCACCA
	3496bp-3882bp	GCAGGTGCAGACAGTAACCAGACCGCAGGTGATCGCAGCAAACTGCTGG	GAGGAGAAGCCCGGTTCACTATTCAACTTCAATCCAATCGTCTTCTT
INpro-28R	1bp-570bp	GACGACGACAAGATGGATTACGACATCCCCACTACTGAGAATCTTTATTTTCAGGGCATGAACCTGGATAAAGCTCTGGT	TTGGGCCGTCTGCGTGCTACCATAACCAGCGGTCAGGTTGCTGCCTTCTTGAGCGGTCTGCGTCGAACCGTAGCCGGCCGTCAG
	2920bp-3882bp	GCTGGTTATGGTAGCACGCAGACGGCCCAAGAAAATTCATCGCTGACGAC	GAGGAGAAGCCCGGTTCACTATTCAACTTCAATCCAATCGTCTTCTT
INpro-67R	1bp -3882bp	GACGACGACAAGATGGATTACGACATCCCCACTACTGAGAATCTTTATTTTCAGGGCATGAATCTCGACAAGGCGTTGGTGCTGC	GAGGAGAAGCCCGGTTCACTACTCTACCTCTATCCAGTCATCTTCCTCGTCGGG
INpro-16R- ΔN	1bp-570bp	GACGACGACAAGATGGATTACGACATCCCCACTACTGAGAATCTTTATTTTCAGGGCGCGACCTACGGCTCTACCCTGAGTGGTG	GTAACCCGCGGTTTGCGTAGAACCATAGCCTGCAATCAG GCGACTATGATTATCACCACTCAGGGTAGAGCC
	3019bp-3882bp	GCAGGCTATGGTTCTACGCAAACCGCGGGT	GAGGAGAAGCCCGGTCACTATTCAACTTCAATCCAATCGTCTTCTT
INpro-15R- ΔT	1768bp-2487bp	GACGACGACAAGATGGATTACGACATCCCCACTACTGAGAATCTTTATTTTCAGGGCGCTGGCTATGGTTCAACCGGCACGGCCGGT	GAGGAGAAGCCCGGTTCACTACGTCAGGATGCTTTTGAAACCCGCGGTTTG
INpro-N-1R	3736bp-3882bp	GACGACGACAAGATGGATTACGACATCCCCACTACTGAGAATCTTTATTTTCAGGGCTTCAGGCTCTGGGACGGGAAG	GAGGAGAAGCCCGGTTCACTACTCTACCTCTATCCAGTCATCTTCCTCGTCGGG
INpro-C	1bp-570bp	GACGACGACAAGATGGATTACGACATCCCCACTACTGAGAATCTTTATTTTCAGGGCATGAATCTCGACAAGGCGTTGGTGCTGC	GAGGAGAAGCCCGGTTCACGGCAATGAGCCGACT

Table S2: An overview of cloning and purification details for all protein constructs used in the study. *AC – affinity chromatography; SEC – size-exclusion chromatography; AEC – Anion exchange chromatography; ASP – Ammonium sulphate precipitation. All strains were expressed in Rosetta (DE3) Competent *E. coli* Cells (Novagen).

**Protein construct**	**Vector**	**Tag**	**Tev site**	**Mol. weight [kDa]**	**Purification steps***	**Detergent in the final sample**
INpro-9R	*pET-30 Ek/LIC*	N-terminal His Tag and S Tag	Y	41	AC, SEC	No detergent
INpro-16R	*pET-30 Ek/LIC*	N-terminal His Tag and S Tag	Y	53,7	AC, SEC	No detergent
INpro-28R	*pET-30 Ek/LIC*	N-terminal His Tag and S Tag	Y	72	AC, SEC	0.05% n-Dodecyl β-D-maltoside (DDM)
INpro-67R	*pET24+*	C-terminal His Tag	N	143.6	AEC, ASP, SEC	0.1% N-lauroylsarcosine (NLS)
INpro-16R- ΔN	*pET-30 Ek/LIC*	N-terminal His Tag and S Tag	Y	37.6	AC, SEC	No detergent
INpro-15R- ΔT	*pET-30 Ek/LIC*	N-terminal His Tag and S Tag	Y	25	AC, SEC	No detergent
INpro-1R-N	*pET-30 Ek/LIC*	N-terminal His Tag	Y	21.6	AC, AEC, SEC	No detergent
INpro-C	*pET-30 Ek/LIC*	N-terminal GST Tag , S tag and His Tag	Y	31.5	AC, AEC, SEC	No detergent

Table S3: Properties of the different protein constructs and oligomerization states. $T_{char,knee}$, $T_{char,50}$, $T_{char,10}$, $T_{char,1}$ are the characteristic nucleation temperature of homogeneous INpro at 100%, 50%, and 10% of the knee point.

**Protein construct**	**Presumed state of oligomerization**	**Length /nm** [a]	**Width /nm** [a]	**INpro class**	$N_{n,\ knee}$ **/n**$^{-1}$	$T_{char,knee}$**/°C**	$T_{char,50}$ **/°C**	$T_{char,10}$ **/°C**	**Concentration [mg/mL]**	**instrument**
INpro-9R	dimer	4.2	5.5	C	2.55×10^{-10}	-24.7	-23.7	-22.3	0.189-0.225	WISDOM
INpro-16R	dimer	7.4	5.5	C	4.44×10^{-7}	-22.2	-21.7	-21.3	0.04-0.47	WISDOM
INpro-28R	dimer	13	5.5	C	2.33×10^{-5}	-16.3	-15.8	-14.2	0.078-0.146	WISDOM
INpro-67R	dimer	31	5.5	C	1.59×10^{-8}	-11.7	-11.2	-9.8	0.157-0.719	WISDOM
INpro-67R	dimer	31	5.5	C					0.143	LINA
	higher-order oligomer	na	na	A						
INpro-16R	dimer	7.4	5.5	C	4.44×10^{-7}	-22.2	-21.7	-21.3	0.3-0.63	LINA
	higher-order oligomer	na	na	A	1.25×10^{-14} [b]	-9.6	-9.3	-9.0		
E.coli expressing INpro-67R	dimer	/	/	C					/	LINA
E.coli expressing INpro-67R	higher-order oligomer	/	/	A	4.04×10^{3} [c]	-4.7	-4.5	-4.1	/	LINA

[a] The legth and the width are derived from the structural INpro model.

[b] Knee point and derived values have higher uncertainties as the data base is low.

[c] Nv instead of Nn
